# Tumor Microenvironment‐responsive Nanocatalyst for Targeted Chemodynamic Cancer Therapy

**DOI:** 10.1002/adhm.202501746

**Published:** 2025-06-17

**Authors:** Jun Ma, Jingjing Qiu, Shiren Wang

**Affiliations:** ^1^ Department of Biomedical Engineering Texas A&M University College Station TX 77843 USA; ^2^ Department of Mechanical Engineering Texas A&M University College Station TX 77843 USA; ^3^ Department of Industrial Systems and Engineering Texas A&M University College Station TX 77843 USA; ^4^ Department of Materials Science and Engineering Texas A&M University College Station TX 77843 USA

**Keywords:** Cancer Therapy, Core/Shell Nanoparticles, Nanocatalyst, Nanomedicine

## Abstract

To address the challenges of insufficient hydrogen peroxide (H_2_O_2_) levels, rapid Fe^3+^ precipitation, and a slow Fenton reaction cycle, tumor‐activated, self‐accelerating CDT nanocatalysts are synthesized, comprising poly (lactic‐co‐glycolic acid) (PLGA)‐encapsulated Ca‐Fe peroxide clusters and polyarginine (R). Nanocatalysts are camouflaged with cancer cell membranes (CCM) to enhance tumor targeting. Additionally, polyarginine tailored the PLGA responsiveness to low H_2_O_2_ levels (50–100 µm). H_2_O_2_ triggered the degradation of PLGA, releasing CaFe clusters to produce Fe^3+^/Fe^2+^ and additional H_2_O_2_, sustaining the Fenton reaction. Simultaneously, polyarginine releases nitric oxide (NO) in the presence of H_2_O_2_, facilitating Fe^3+^ reduction to Fe^2+^ and amplifying •OH generation. In vitro cellular studies demonstrate significantly improved homotypic tumor targeting (6.5‐fold increase) and deep spheroid penetration (>120 µm), resulting in improved tumor permeability and elevated •OH generation. Additionally, the nanoparticles exhibit dose‐dependent cytotoxicity, and polyarginine notably enhanced the cytotoxicity of CCM‐PLGA‐CaFe NPs, reducing the IC50 value from 216.9 to 43.38 µg mL^−1^. Apoptosis/necrosis assay reveals that the elevated •OH generation by CCM‐PLGA‐CaFe‐R NPs preferentially induced necrosis, effectively inhibiting tumor cell proliferation by 76.3% ± 8.4% over a 7‐day treatment. Consequently, this TME‐responsive, self‐accelerating CDT platform demonstrates enhanced therapeutic efficacy through improved tumor targeting, sustained Fenton reaction, and amplified radical generation.

## Graphical abstract

An Tumor microenvironment‐responsive nanocatalyst system composed of PLGA‐encapsulated Ca–Fe peroxide and polyarginine enables sustainable Fenton reaction in tumors. Low levels of H_2_O_2_ (50–100 µm) initiate the nanoparticle degradation, while polyarginine‐derived nitric oxide accelerates Fe^3+^ reduction, leading to self‐amplifying **•**OH generation for enhanced chemodynamic cancer therapy.

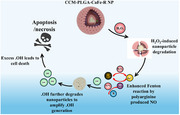

## 1 Introduction

Cancer cells adapt to rapid proliferation by altering their metabolism and protein translation, resulting in elevated reactive oxygen species (ROS) levels that drive cellular changes and foster tumor growth.^[^ ^1^ ^]^ Cancer cells upregulate antioxidant systems to maintain ROS levels below the cytotoxic threshold while exploiting elevated basal ROS to survive in this oxidative environment.^[^ ^2^ ^]^ However, this adaptation renders cancer cells more susceptible to additional oxidative stress than normal cells.^[^ ^3^, ^4^ ^]^ Leveraging this vulnerability, therapies such as photodynamic therapy (PDT), sonodynamic therapy (SDT), and chemodynamic therapy (CDT), have been developed to amplify ROS production within tumors. Among these ROS, hydroxyl radicals (•OH) are highly cytotoxic, generated through Fenton or Fenton‐like reactions between hydrogen peroxide (H_2_O_2_) and catalytic metals. However, traditional Fe‐based Fenton agents (e.g., Fe_3_O_4_,^[^ ^5^, ^6^ ^]^ FeS,^[^ ^7^ ^]^ FeS_2_,^[^ ^8^, ^9^ ^]^ and Fe_2_P^[^ ^10^ ^]^) require strong acidity (pH 3–4) to initiate these reactions, limiting their efficacy in the mildly acidic tumor microenvironment (TME).^[^ ^11^ ^]^ Moreover, CDT is constrained by a slow Fenton reaction cycle, characterized by the rapid precipitation of Fe^3+^ at neutral pH and its sluggish reduction back to Fe^2+^,^[^ ^12^, ^13^ ^]^ coupled with insufficient H_2_O_2_ levels (50–100 µm) within the tumor microenvironment(TME).

Although emerging Fenton catalysts, such as Mn‐based,^[^ ^14^ ^]^ Mo‐based agents,^[^ ^15^, ^16^, ^17^ ^]^ and peroxidase mimics (e.g., carbon dots^[^ ^18^ ^]^), exhibit broader pH adaptability, they are still hampered by insufficient H_2_O_2_ availability in the TME. Recent strategies to supply sufficient H_2_O_2_ include stimulating in situ generation by disrupting the TME's redox balance or introducing exogenous sources of H_2_O_2_, such as metal peroxides (e.g., CuO_2_,^[^ ^19^ ^]^ CaO_2_^[^ ^20^ ^]^). In situ generation of H_2_O_2_ can be enhanced by promoting the conversion of superoxide ions within TME into H_2_O_2_ and reducing its elimination (e.g., inhibition of catalase),^[^ ^21^ ^]^ However, these approaches require complex nanocatalyst designs and may still result in inadequate H_2_O_2_. Alternatively, self‐supplying metal peroxides, such as CuO_2_,^[^ ^19^ ^]^ CaO_2_,^[^ ^20^ ^]^ and hybrid metal peroxides (Cu‐Fe,^[^ ^22^ ^]^ Cu–Ca,^[^ ^23^ ^]^ Cu–Ru^[^ ^24^ ^]^), can provide ample H_2_O_2_. However, these metal peroxides pose safety concerns due to potential premature leakage and toxic metal ion release in physiological environments.^[^ ^21^ ^]^ Thus, stable and TME‐selective metal peroxide systems are needed to maximize ROS production while maintaining systemic stability.

To further enhance the CDT, it is crucial to accelerate the Fenton cycle by stabilizing Fe^3+^ and promoting its reduction. Chelation strategies using poly‐ligands can prevent Fe^3+^ precipitation and sustain Fe^2+^ regeneration. Tannic acid (TA) is a notable molecule capable of serving as a chelate and a reductive agent. While it can chelate Fe^3+^ ions to boost CDT,^[^ ^25^, ^26^, ^27^ ^]^ it also acts as a potent radical scavenger, particularly for H_2_O_2_ and •OH,^[^ ^28^, ^29^ ^]^ undermining the therapeutic efficacy. Acid‐terminated poly(lactic‐co‐glycolic) acid (PLGA) presents a more promising alternative due to its multiple carboxylic groups, biocompatibility, and suitability for nanoparticle modification.^[^ ^30^ ^]^ PLGA not only enhances Fe^3+^ availability but also serves as a protective coating to reduce premature leakage of metal peroxides. Developing novel reductive molecules that can accelerate the Fenton reaction cycle with minimal interference with •OH remains crucial for effective CDT.

In this context, nitric oxide (NO) has emerged as a potential Fenton cycle enhancer. NO has a high affinity for hemoglobin and can preferentially reduce ferric hemoglobin (Fe^3+^) to its ferrous form (Fe^2+^) at low concentrations.^[^ ^31^ ^]^ Studies have shown that NO can boost the Fenton reaction by reducing Fe^3+^ into Fe^2+^ in chemical systems^[^ ^32^ ^]^ and microorganisms (e.g., *E. coli*).^[^ ^33^, ^34^, ^35^ ^]^ Additionally, NO‐mediated cytotoxicity in human ovarian cancer cells has been linked to enhanced Fenton reactions through Fe^3+^ reduction or catalase inhibition, and stabilizing H_2_O_2_.^[^ ^32^ ^]^ Despite its promise, the direct role of NO in Fenton chemistry within the TME remains largely unexplored.

**Scheme 1:**
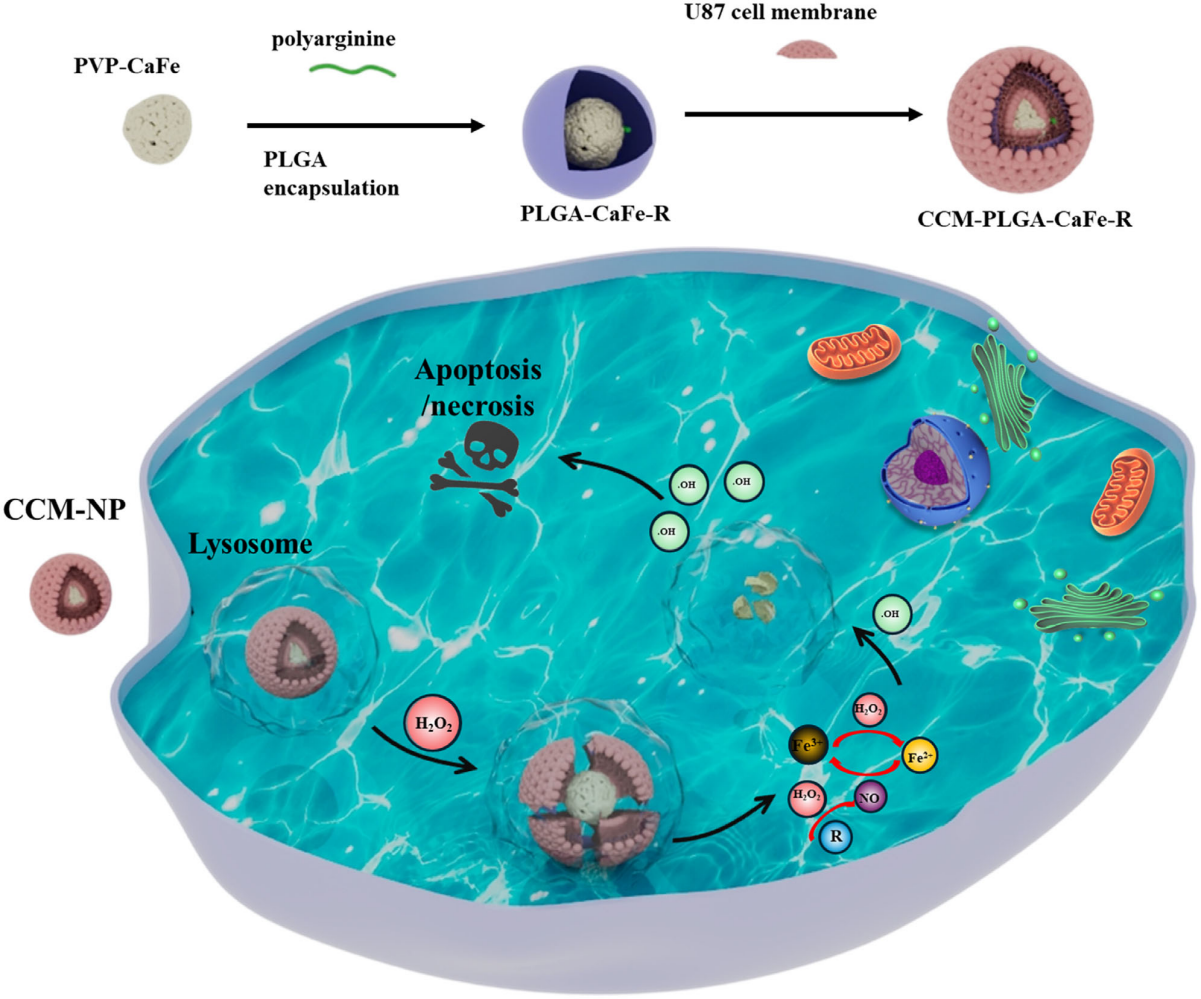
Illustration of nanocatalyst design and self‐accelerating Fenton reaction for effective tumor therapy.

To tackle these challenges, we engineered a TME‐responsive nanoparticle system integrating PLGA‐encapsulated CaFe NPs with polyarginine (R) for enhanced CDT (**Scheme** **1**). Coated with CCM, these nanoparticles can specifically target tumor cells, and polyarginine confers ROS responsiveness to conventionally inert PLGA. Under physiological conditions, the PLGA shell remains stable, while within the TME, H_2_O_2_ degrades PLGA in the presence of polyarginine, releasing CaFe NPs and producing Fe^2+^ and H_2_O_2_. This degradation triggers the Fenton reaction, generating •OH and degrading PLGA, leading to a self‐accelerating reaction cycle. Concurrently, polyarginine produces NO upon H_2_O_2_ exposure, accelerating Fe^3+^ reduction to Fe^2+^ and amplifying •OH generation. This novel TME‐responsive, self‐accelerating platform presents an efficient and targeted approach for efficient CDT.

## 2 Results and Discussion

### 2.1 Nanoparticle Synthesis and Characterizations

**Figure 1:**
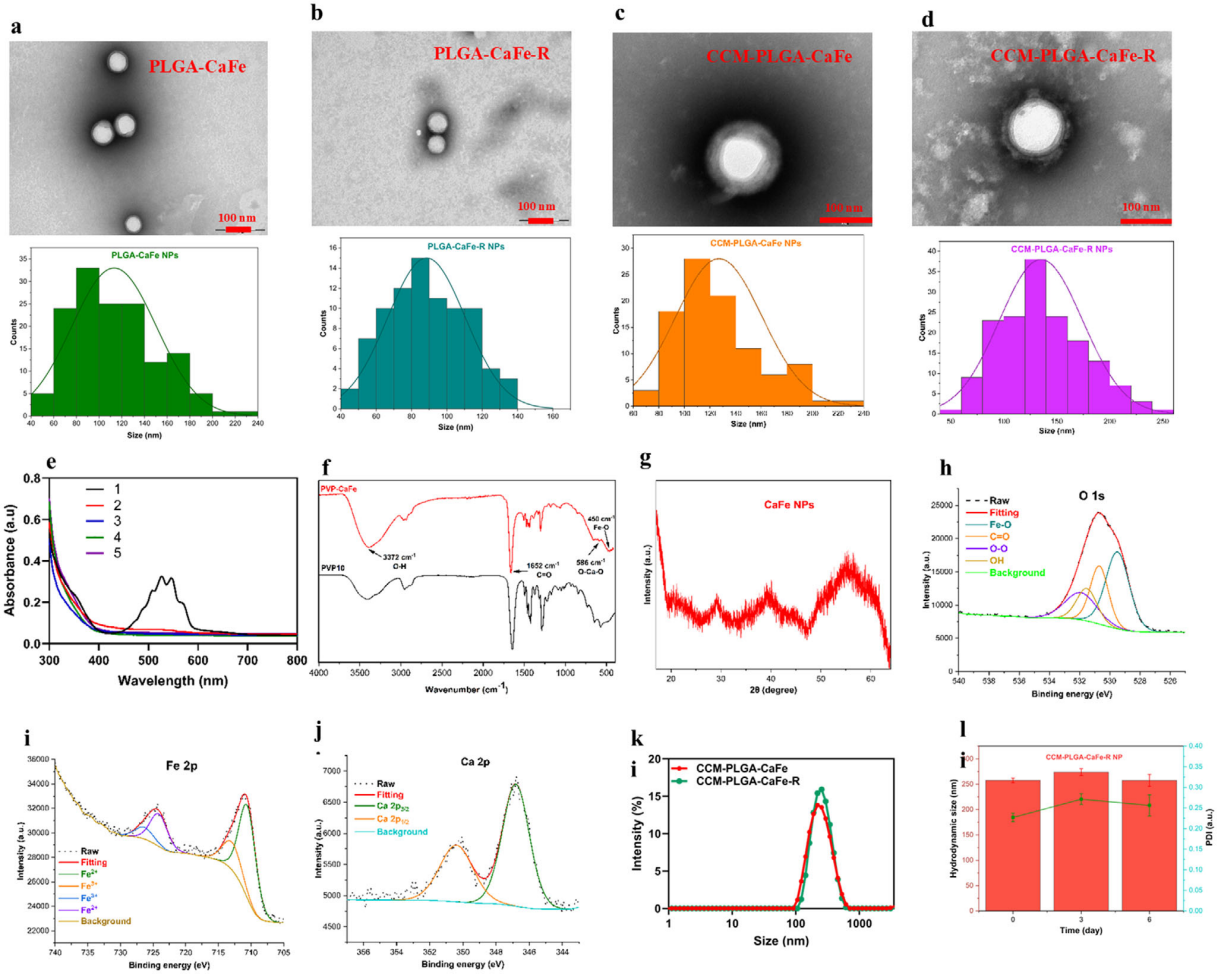
Characterizations of synthesized nanoparticles. TEM images and size distributions of a) PLGA‐CaFe NPs, b) PLGA‐CaFe‐R NPs, c) CCM‐PLGA‐CaFe NPs, and d) CCM‐PLGA‐CaFe‐R NPs. e) UV‐vis scanning of KMnO_4_ solutions after overnight incubation with various CaFe solutions: (1) 0.2 mm KMnO_4_ solution; (2) 0.2 mm KMnO_4_ + 100 µg mL^−1^ CaFe; (3) 0.2 mm KMnO_4_ + 200 µg mL^−1^ CaFe; (4) 0.2 mm KMnO_4_ + 400 µg mL^−1^ CaFe; (5) 0.2 mm KMnO_4_ + 1000 µg mL^−1^ CaFe; f) FTIR and g) XRD analysis of CaFe. High‐resolution spectra of h) O 1s, i) Fe 2p, and j) Ca 2p of CaFe clusters. k) DLS profiles of CCM‐PLGA‐CaFe NPs and CCM‐PLGA‐CaFe‐R NPs. l) Average hydrodynamic size and corresponding PDI of CCM‐PLGA‐CaFe‐R NPs in DPBS over 6 days (*n* = 3).

As shown in Figure S1 (Supporting Information), the synthesized CaFe formed clusters with some aggregation in DI water. However, after PLGA encapsulation, the stability of CaFe clusters significantly improved, remaining stable after 7 days. Transmission electron microscopy (TEM) analysis revealed that PLGA‐CaFe NPs exhibited a spherical morphology with an average size of ≈113 nm (**Figure** **1**a). Following polyarginine loading, the nanoparticles retained a consistent morphology with a reduced average size of ≈88 nm (Figure 1b). Consistent with a previous study,^[^ ^36^ ^]^ this size reduction is likely due to the electrostatic interactions between the positively charged polyarginine and the negatively charged carboxyl groups, leading to a more compact polymer network. The tighter structure may undergo less deformation upon dehydration, contributing to the smaller size of PLGA‐CaFe‐R NPs observed under TEM.

The cancer cell membrane (CCM) camouflaging created a layered structure, with an average size of ≈127 nm for CCM‐PLGA‐CaFe NPs and ≈135 nm for CCM‐PLGA‐CaFe‐R NPs (Figure 1c,d). Previous studies have indicated that most biomimetic nanoparticles are partially coated,^[^ ^37^, ^38^ ^]^ and negative stains can produce a layered structure around nanoparticles, particularly polymeric nanoparticles, potentially creating artifacts under TEM, leading to misinterpretation of the CCM coating.^[^ ^39^ ^]^ The additional layers observed in the TEM images of CCM‐coated nanoparticles suggest successful CCM coating, further supported by the size distribution analysis.

To validate the presence of peroxo groups in the synthesized CaFe clusters, a KMnO_4_‐based colorimetric method was used. Under acidic conditions, MnO_4_^−^ is reduced by peroxo groups to colorless Mn^2+^, serving as a reliable indicator. After overnight incubation of CaFe clusters with KMnO_4_, the solutions became colorless (Figure S2, Supporting Information), and the disappearance of the characteristic KMnO_4_ absorbance peaks at 526 and 546 nm further confirmed the presence of peroxo groups in the CaFe clusters (Figure 1e).

**Figure 2:**
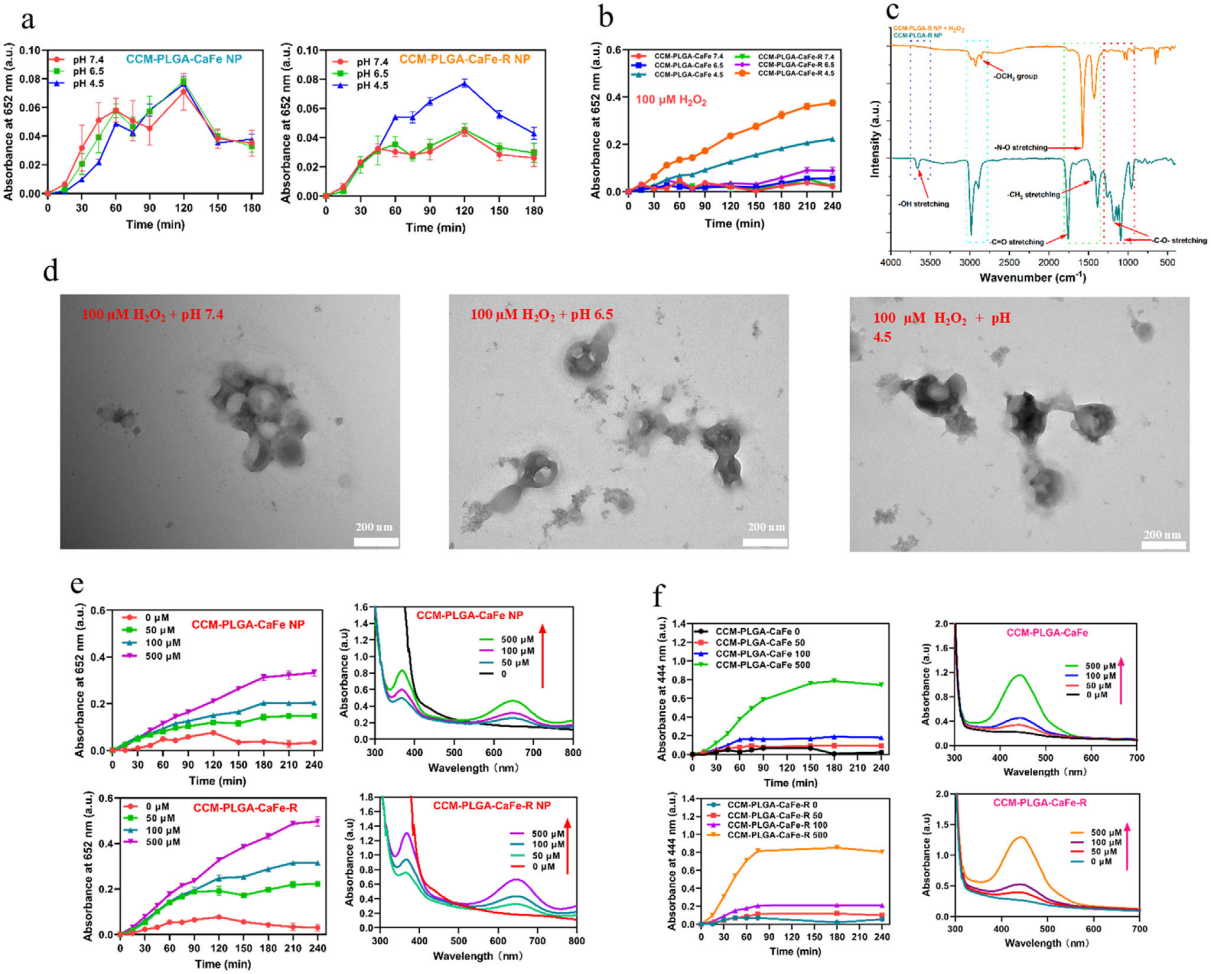
In vitro evaluations of nanoparticle degradation. a) Nanoparticle degradation‐catalyzed oxidation of TMB at different pHs (*n* = 3). b) Nanoparticle degradation‐catalyzed oxidation of TMB at different pHs with 100 µm H_2_O_2_ (*n* = 3). c) FTIR analysis of CCM‐PLGA‐R NPs incubated with and without H_2_O_2_. d) TEM images of PLGA‐CaFe‐R NPs incubated with 100 µm H_2_O_2_ for 2 h at different pH values. Degradation‐catalyzed oxidation of e) TMB and f) OPD with 50, 100, and 500 µm H_2_O_2_ and their UV‐vis absorption spectra (*n* = 3).

To further confirm the successful synthesis of PVP‐CaFe clusters, Fourier Transform Infrared Spectroscopy (FTIR), X‐ray crystallography (XRD), and X‐ray photoelectron spectroscopy (XPS) analyses were performed. In Figure 1f, FTIR analysis revealed a strong absorption peak at ≈1652 cm^−1^, corresponding to the C═O stretching and indicating the presence of PVP on the surface of CaFe clusters. Peaks at ≈586 and 450 cm^−1^ correspond to O─Ca─O^[^ ^20^ ^]^ and Fe─O stretching,^[^ ^40^ ^]^ respectively. X‐ray powder diffraction (XRD) pattern displayed characteristic peaks for CaO_2_ and iron oxide, with peaks at 2*θ* = 29.164 and 55.454° corresponding to CaO_2_,^[^ ^41^ ^]^ and a peak at 2*θ* = 39.275° associated with hydrated iron oxide,^[^ ^42^ ^]^ confirming the coexistence of CaO_2_ and iron oxide lattices in CaFe clusters (**Figure** **2**g).

X‐ray photoelectron spectroscopy (XPS) further examined the surface chemical composition of CaFe clusters. The full survey spectra (Figure S3, Supporting Information) identified Fe, O, and Ca as primary elements, along with C and N. As shown in Figure 1h, high‐resolution O1s spectra revealed peaks at 529.5, 530.7, 531.5, and 532 eV, corresponding to Fe─O, C═O, OH, and O─O groups, respectively, confirming the presence of PVP, Fe, and peroxo groups on the surface of CaFe clusters.^[^ ^43^, ^44^ ^]^ Since Fe cannot coordinate with (O_2_)^2−^ to form metal peroxides,^[^ ^45^ ^]^ the O─O groups are likely present in CaO_2_, further supported by O─Ca─O stretching in FTIR spectra. The Fe 2p spectra indicated the coexistence of Fe^2+^ and Fe^3+^, with peaks at 710.5 and 724.1 eV assigned to Fe^2+^ and peaks at 712.9 and 726.3 eV attributed to Fe^3+^ (Figure 2i).^[^ ^46^ ^]^ The calculated Fe^2+^/Fe^3+^ ratio was 2.84, showing minimal oxidation of Fe^2+^ during synthesis. Additionally, Ca 2p spectra exhibited two characteristic peaks at 346.8 and 350.4 eV, suggesting the presence of Ca^2+^ on the cluster surface (Figure 2j).^[^ ^47^, ^48^ ^]^

After coating with CCM, the hydrodynamic size of nanoparticles was evaluated using dynamic light scattering (DLS). The hydrodynamic diameter of CCM‐PLGA‐CaFe‐R NPs increased to 257 from 240 nm of CCM‐PLGA‐CaFe NPs (Figure 1k). To assess the aqueous stability of CCM‐PLGA‐CaFe‐R NPs, the hydrodynamic size of nanoparticles was monitored in DPBS over 6 days. As shown in Figure 1l, CCM‐PLGA‐CaFe‐R NPs maintained stable size throughout the 6 days, consistent with the observed changes in polydispersity index (PDI), indicating superior stability of CCM‐PLGA‐CaFe‐R NPs in physiological fluids.

Before other assays, the encapsulated CaFe content in PLGA‐CaFe NPs and PLGA‐CaFe‐R NPs was determined using a thermogravimetric analyzer (TGA). The mass changes of CaFe cluster, PLGA NPs, PLGA‐R NPs, PLGA‐CaFe NPs, and PLGA‐CaFe‐R NPs were monitored over an 800 °C temperature change (Figure S4, Supporting Information). Further analysis indicated that CaFe content in PLGA‐CaFe NPs was 12.4% while polyarginine (R) loading decreased the CaFe content in PLGA‐CaFe‐R NPs to 9.3%.

### 2.2 TME‐Responsive Degradation and Hydroxyl Radical Production

The tumor microenvironment (TME) is characterized by mild acidity and elevated levels of hydrogen peroxide (H_2_O_2_, 50–100 µm).^[^ ^49^ ^]^ Colorimetric characterizations were employed to evaluate the pH effects on nanoparticle degradation. Upon degradation of PLGA‐CaFe NPs and PLGA‐CaFe‐R NPs, the produced H_2_O_2_ reacts with Fe^2+^ to generate hydroxyl radical (**•**OH), which converts colorless dyes TMB and OPD into their colored oxidized forms, blue oxTMB, and yellow oxOPD, respectively. The degradation of PLGA‐CaFe‐R NPs was accelerated by acidic pH (6.5 and 4.5) while no pH‐dependence was observed for the degradation of PLGA‐CaFe NPs (Figure S5a, Supporting Information). After CCM coating, a similar trend for pH‐dependent degradation was observed (Figure 2a).

In 100 µM H_2_O_2_, nanoparticle degradation was accelerated under acidic conditions. At neutral pH 7.4, only minimal changes in absorbance were observed. At pH 6.5, both PLGA‐CaFe‐R NPs and CCM‐PLGA‐CaFe‐R NPs had slightly faster degradation than PLGA‐CaFe NPs and CCM‐PLGA‐CaFe NPs, respectively (Figure 2b; Figure S5b, Supporting Information). Notably, at pH 4.5, H_2_O_2_ significantly enhanced the degradation of both nanoparticles, as evidenced by a much stronger absorbance over time compared to nanoparticles without H_2_O_2_. These results suggest that H_2_O_2_ may initiate the nanoparticle degradation, with acidic pH facilitating the Fenton reaction activity and subsequent **•**OH production.

Study demonstrates that the peroxyl radicals, generated by e‐beam irradiation under oxygenated conditions, can cause the chain scission of PLGA.^[^ ^50^ ^]^ However, it remains unclear whether the nanoparticle degradation is driven explicitly by the H_2_O_2_‐induced Fenton reaction, resulting in **•**OH production, or if H_2_O_2_ alone is sufficient to degrade PLGA NPs directly. FTIR analysis (Figure S6a, Supporting Information) showed that 100 µm H_2_O_2_ alone caused no detectable changes in PLGA, whereas adding Fe^2+^ ions led to the disappearance or reduction of several characteristic peaks, indicating that 100 µm H_2_O_2_ alone is insufficient to degrade PLGA, while **•**OH radicals generated through the Fenton reaction can effectively degrade PLGA.

The effect of 100 µm H_2_O_2_ on PLGA‐R NPs and CCM‐PLGA‐R NPs was also examined. For PLGA‐R NPs, H_2_O_2_ treatment led to the disappearance/reduction of peaks at 1760 cm^−1^ (C═O stretching), 1467 cm^−1^ (CH_3_ bending), 1348 cm^−1^ (CH bending), 1307 cm^−1^ (CH stretching), and 1128 cm^−1^ (CH_3_ antisymmetric rocking), confirming degradation (Figure S6, Supporting Information). Similarly, in CCM‐PLGA‐R NPs (Figure 2c), peaks at 3663 cm^−1^ (‐OH stretching), 1756 cm^−1^ (C═O stretching), 1456 cm^−1^ (CH_2_ stretching), and 1181/1091 cm^−1^ (C─O stretching) vanished following H_2_O_2_ exposure.^[^ ^51^ ^]^ The emergence of new peaks at 1574 cm^−1^ (N‐O stretching) and 2856 (‐OCH_2_ group) suggested the formation of degradation products.^[^ ^52^ ^]^ These findings show that PLGA alone is unresponsive to H_2_O_2_, while polyarginine confers H_2_O_2_ responsiveness, tailoring PLGA nanoparticles to degrade under low H_2_O_2_ levels.

Further TEM analysis revealed that H_2_O_2_ compromised the structural integrity of PLGA‐CaFe‐R NPs at pH 7.4, 6.5, and 4.5, leading to the CaFe leakage and confirming the responsiveness of nanoparticles to H_2_O_2_ (Figure 2d). The effect of H_2_O_2_ concentrations on nanoparticle degradation was also assessed by monitoring the color changes in TMB and OPD. As shown in Figure S7 (Supporting Information), PLGA‐CaFe‐R NPs were incubated with 50 and 500 µm H_2_O_2_ at pH 4.5; the absorbance intensity of oxTMB and oxOPD increased with higher H_2_O_2_ concentrations compared to the control group (H_2_O_2_ + dye), implying accelerated degradation and enhanced **•**OH production. After CCM coating, nanoparticles retained comparable H_2_O_2_ responsiveness (Figure 2e,f) using both TMB and OPD as indicators.

**Figure 3:**
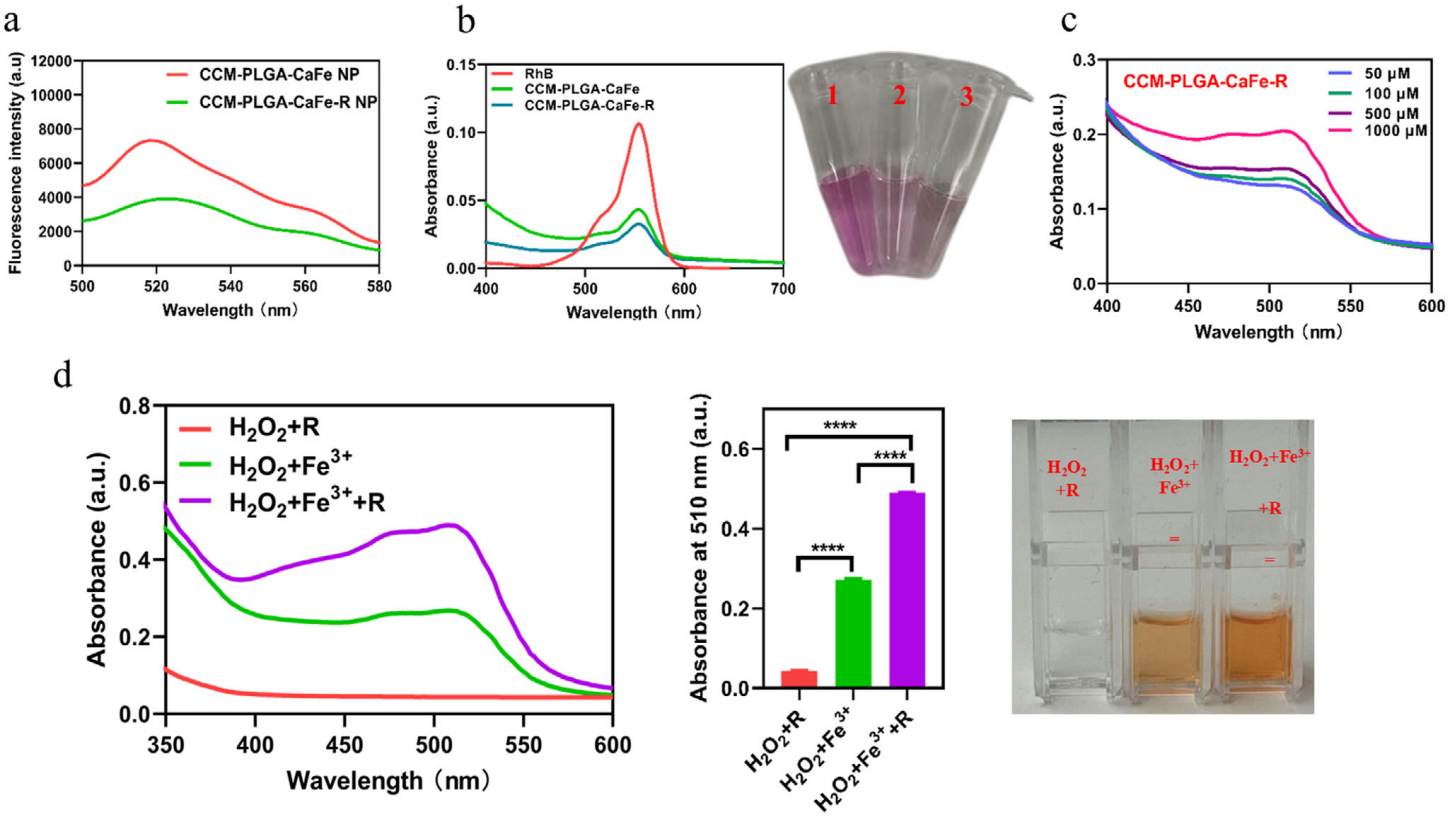
In vitro assessment of the hydroxyl radical (**•**OH) production and Fe^2+^ availability of nanoparticles. a) **•**OH production detected by the fluorescence of APF at pH 6.5 with 100 µm H_2_O_2_. b) Decolorization assay nanoparticles using Rhodamine B at pH 6.5 with 100 µm H_2_O_2_. c) Fe^2+^ availability of CCM‐PLGA‐CaFe‐R NPs at different pH and different concentrations of H_2_O_2_. d) UV‐vis spectra (left), absorbance quantification (middle), and representative images of Fe^2+^ reduced from Fe^3+^ by polyarginine (right). Data were analyzed with One‐way ANOVA (n = 3). **** *p* < 0.0001.

APF probe was used to evaluate hydroxyl radical (**•**OH) production during nanoparticle degradation. Figure S8 (Supporting Information) shows that PLGA‐CaFe‐R NPs generated more **•**OH than PLGA‐CaFe NPs at acidic pH (6.5 and 4.5). Likewise, **Figure** **3**a demonstrates that at pH 6.5 with 100 µm H_2_O_2_, CCM‐PLGA‐CaFe‐R NPs exhibited higher fluorescence intensity than CCM‐PLGA‐CaFe NPs, confirming polyarginine's enhancement of **•**OH generation. A Rhodamine B (RhB) decolorization assay further supported this: In the presence of 100 µM H_2_O_2_, both CCM‐PLGA‐CaFe NPs and CCM‐PLGA‐CaFe‐R NPs reduced RhB absorbance (Figure 3b), providing additional evidence of **•**OH formation.

To verify the enhancement of Fe^2+^ availability by polyarginine, a colorimetric detection assay using 1,10‐phenanthroline (phen), which forms a red‐orange Fe^2+^ complex with an absorbance peak at 510 nm, was performed. As shown in Figure S9 (Supporting Information), initial tests on pH effects showed minimal impact on Fe^2+^ availability in PLGA‐CaFe NPs. In contrast, acidic pHs slightly increased the Fe^2+^ availability in PLGA‐CaFe‐R NPs. Further investigation with H_2_O_2_ revealed that Fe^2+^ levels increased with higher H_2_O_2_ concentrations for both PLGA‐CaFe NPs and PLGA‐CaFe‐R NPs. This effect was more pronounced in PLGA‐CaFe‐R NPs, highlighting the role of polyarginine in converting Fe^3+^ to Fe^2+^. CCM‐PLGA‐CaFe‐R NPs also retained H_2_O_2_‐dependent Fe^2+^ availability (Figure 3c).

In a straightforward Fenton reaction (Fe^3+^+H_2_O_2_), adding polyarginine remarkably facilitated Fe^3+^ reduction to Fe^2+^, yielding higher Fe^2+^ concentrations in Fe^3+^+H_2_O_2_+R solution (Figure 3d). These results demonstrate that CCM‐PLGA‐CaFe‐R NPs can serve as a TME‐responsive therapeutic, with the TME driving degradation and enhancing the Fenton reaction for efficient **•**OH production.

### 2.3 In Vitro Cellular Uptake and Evaluation of Enhanced CDT

**Figure 4:**
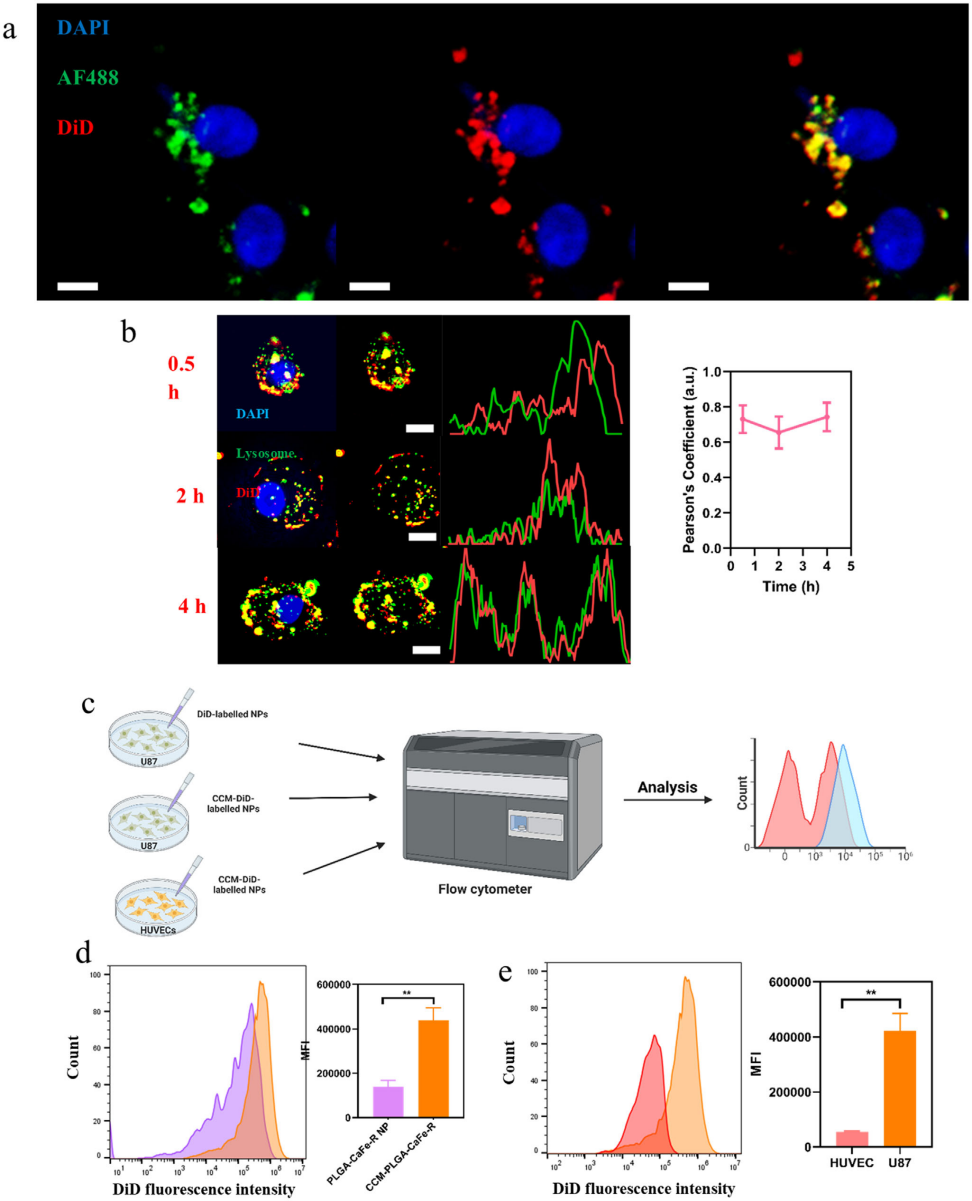
In vitro cellular uptake of nanoparticles. Scale bar: 20 µm. a) Colocalization of CCM and polymer core of CCM‐PLGA‐CaFe‐R NPs upon cellular uptake. b) Co‐localization of lysosomes and DiD‐labeled CCM‐PLGA‐CaFe‐R NPs at 0.5, 2, and 4 h. Rectangular scan profiles were analyzed by ImageJ. c) Scheme of cellular uptake assay analyzed by flow cytometry Flow cytometric analysis of cellular uptake of CCM‐PLGA‐CaFe‐R‐DiD NPs and corresponding mean intensity of cellular DiD fluorescence intensity in U87 cells and HUVECs. d) Flow cytometric analysis of cellular uptake of PLGA‐CaFe‐R‐DiD and CCM‐PLGA‐CaFe‐R‐DiD NPs and corresponding mean intensity of cellular DiD fluorescence intensity in U87 cells. e) Flow cytometric analysis of cellular uptake of CCM‐PLGA‐CaFe‐R‐DiD NPs and corresponding mean intensity of cellular DiD fluorescence intensity in U87 cells and HUVECs. Statistical significance was analyzed with one‐sided Student *t*‐test (*n* = 3). **p < 0.01.

To assess the integrity of CCM during cellular uptake, we used a dual‐fluorophore‐labeled CCM‐PLGA‐CaFe‐R NPs, in which the polymer core was labeled with Alexa Fluor. In the inr 488 dye contrast, CCM was labeled with DiD dye. After 2‐h incubation with U87 cells, strong colocalization between the core and CCM fluorescence signals was observed (**Figure** **4**a). Consistent with previous studies,^[^ ^53^, ^54^ ^]^ this high degree of overlap suggests that the CCM coating remains intact during uptake and that the nanoparticles are internalized primarily via endocytosis rather than membrane fusion.

DiD‐labeled nanoparticles were incubated with U87 cells for different times to determine the subcellular distribution further. The endocytosis of these nanoparticles was assessed using LysoBrite Green, a specific dye for labeling endosomes/lysosomes. As illustrated in Figure 4b, following internalization, the nanoparticles accumulated in lysosomes and remained co‐localized with lysosomes after 4 h, as evidenced by overlapping rectangular scan profiles and consistently high Pearson's coefficients over time. This lysosomal accumulation is critical for efficient production of **•**OH, which relies on an acidic pH environment.

To demonstrate the specific targeting ability of cancer cell membrane‐camouflaged nanoparticles (CCM‐NPs) toward glioblastoma cells, cellular uptake assays were performed using DiD‐labeled NPs and analyzed by flow cytometry (Figure 4c). As shown in Figure 4d, flow cytometry analysis revealed that the uptake of CCM‐PLGA‐CaFe‐R‐DiD NPs by U87 cells was three times that of PLGA‐CaFe‐R‐DiD NPs (mean DiD fluorescence intensity of 423,017 versus 139460), indicating enhanced tumor targetability due to CCM. Additionally, the uptake of CCM‐PLGA‐CaFe‐R‐DiD NPs by U87 cells was approximately 6.7 times greater than that by HUVECs (mean DiD fluorescence intensity of 423,017 versus 54758), underscoring the cell selectivity of CCM‐PLGA‐CaFe‐R‐DiD NPs (Figure 4e). Further analysis of other nanoparticles, including CCM‐PLGA‐DiD NPs, CCM‐PLGA‐R‐DiD NPs, and CCM‐PLGA‐CaFe‐DiD NPs, also supports the tumor selectivity of CCM‐coated nanoparticles (Figure S10, Supporting Information).

**Figure 5:**
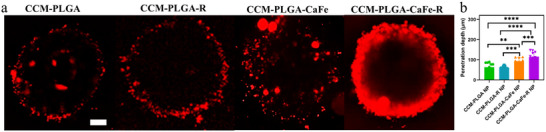
In vitro evaluations of nanoparticle penetration into tumor spheroids. a) Central cross‐section U87 spheroids penetrated by DiD‐labelled nanoparticles. b) Mean penetration depth of various nanoparticles. Scale bar: 100 µm. Data were analyzed with One‐way ANOVA (n = 4). ** *p* < 0.01, *** *p* < 0.001, **** *p* < 0.0001.

Following confirming the cellular uptake of CCM‐NPs in U87 cells, we investigated nanoparticle interactions with the tumor spheroids to mimic the tumor microenvironment (TME) closely, as shown in Figure 5. Large spheroids (>400–500 µm) exhibit a layered structure, with a necrotic core surrounded by viable quiescent and proliferating cells, creating physicochemical gradients (e.g., pH, H_2_O_2_, O_2_, and nutrition).^[^ ^55^, ^56^ ^]^ We first assessed the penetration and distribution of CCM‐NPs within spheroids, as penetration of nanoparticles determines the overall therapeutic efficacy in solid tumors.^[^ ^57^ ^]^ Laser scanning confocal microscopy (LSCM) analysis showed that CCM‐PLGA and CCM‐PLGA‐R NPs mainly accumulated at the spheroid periphery. In contrast, CCM‐PLGA‐CaFe NPs and PLGA‐CaFe‐R NPs penetrated deeper. Remarkably, CCM‐PLGA‐CaFe‐R NPs achieved the deepest penetration, with an average depth of 128.9 ± 25.6 µm into the spheroid core (**Figure** **5**).

**Figure 6:**
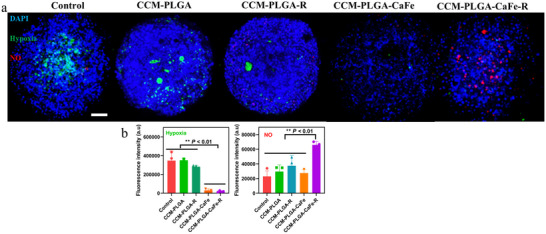
In situ generation of O_2_ and NO in U87 spheroids. a) Hypoxia and NO staining of U87 spheroids with different nanoparticle treatments. Hypoxia and NO were stained by ImageIT Green hypoxia reagent and DAR‐1, respectively. Cell nuclei were stained by DAPI. b) Corresponding mean fluorescence intensity of hypoxia and NO dye with various nanoparticle treatments. Statistical analysis was performed with One‐way ANOVA (*n* = 4). ** *p*< 0.01. Scale bar: 100 µm.

As PLGA‐CaFe‐R NPs degrade, they generate oxygen (O_2_) and hydrogen peroxide (H_2_O_2_), with H_2_O_2_ further reacting with polyarginine to produce nitric oxide (NO). The in‐situ generation of O_2_ and NO within spheroids was assessed using ImageIT Green hypoxia reagent and DAR‐1, respectively. ImageIT Green fluorescence decreases in oxygenated conditions, while DAR‐1 fluorescence increases with higher NO concentrations. As shown in **Figure** **6**, both CCM‐PLGA‐CaFe NPs and CCM‐PLGA‐CaFe‐R NPs significantly reduced hypoxia, as evidenced by decreased fluorescence intensity. Additionally, PLGA‐CaFe‐R NPs notably induced NO generation, as indicated by increased DAR‐1 fluorescence intensity, highlighting the potential of CCM‐PLGA‐CaFe‐R NPs to alleviate hypoxia and generate NO in the TME.

**Figure 7:**
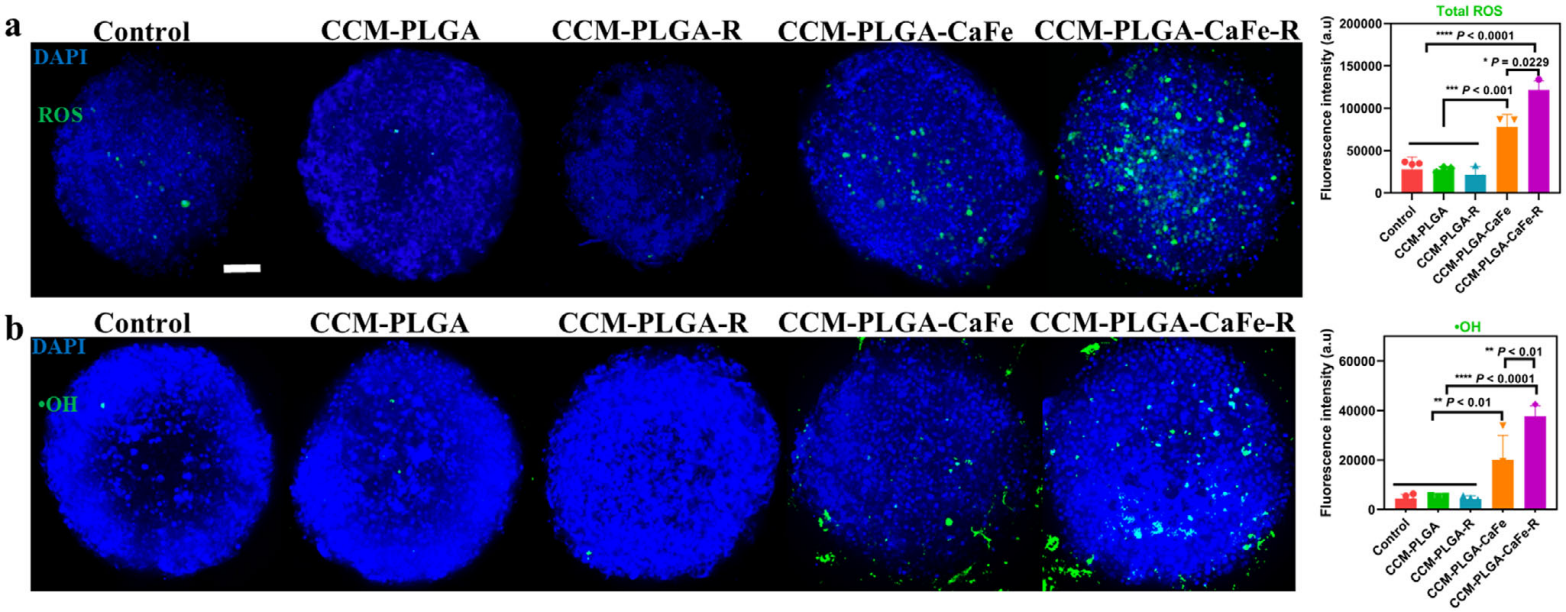
In situ generation of total ROS and **•**OH in U87 spheroids. a) Total ROS generation in U87 spheroids with different nanoparticle treatments. Total ROS and **•**OH were stained by DCFH‐DA and APF, respectively. Cell nuclei were stained by DAPI. b) Corresponding mean fluorescence intensity of hypoxia and NO dye with various nanoparticle treatments. Statistical analysis was performed using One‐way ANOVA (*n* = 4). * *p*< 0.05, ** *p*< 0.01, *** *p*< 0.001, **** *p*< 0.0001. Scale bar: 100 µm.

To evaluate in situ total ROS and **•**OH levels within spheroids, fluorescent staining was performed with the 2′,7′‐dichlorofluorescein diacetate (DCFH‐DA) probe for total ROS and the APF probe for **•**OH. Spheroids treated with both PLGA‐CaFe NPs and PLGA‐CaFe‐R NPs showed significantly higher ROS production, with PLGA‐CaFe‐R NPs yielding ≈4.4 times the fluorescence intensity of control and PLGA NPs, and ≈3.6 times higher than PLGA‐R NPs. Further specific detection of **•**OH followed a similar trend, with elevated **•**OH production observed in both CCM‐PLGA‐CaFe NP and CCM‐PLGA‐CaFe‐R NP‐treated groups compared to other groups. The fluorescence intensity in the CCM‐PLGA‐CaFe‐R NP group was ≈ 1.9 times that of the CCM‐PLGA‐CaFe NP group. These results indicate that CCM‐PLGA‐CaFe NPs and CCM‐PLGA‐CaFe‐R NPs can effectively produce ROS within tumor spheroids, with polyarginine enhancing ROS and **•**OH generation (**Figure** **7**).

To investigate the anti‐tumor effects of produced **•**OH, a short‐term cytotoxicity assay was conducted in 2D cell culture using RFP‐expressing HUVECs and GFP‐expressing U87 cells, with cell viability normalized by RFP and GFP fluorescence intensity, respectively. After 24 h of incubation, up to 1000 µg mL^−1^, CCM‐PLGA and CCM‐PLGA‐R NPs showed no cytotoxicity in both HUVECs and U87 cells. No cytotoxicity was observed below 600 µg mL^−1^ for all HUVECs nanoparticles, while CCM‐PLGA‐CaFe NPs and CCM‐PLGA‐CaFe‐R NPs exhibited dose‐dependent cytotoxicity in U87 cells. Notably, CCM‐PLGA‐CaFe‐R NPs showed greater inhibition of cell proliferation than CCM‐PLGA‐CaFe NPs (Figure S11, Supporting Information).

**Figure 8:**
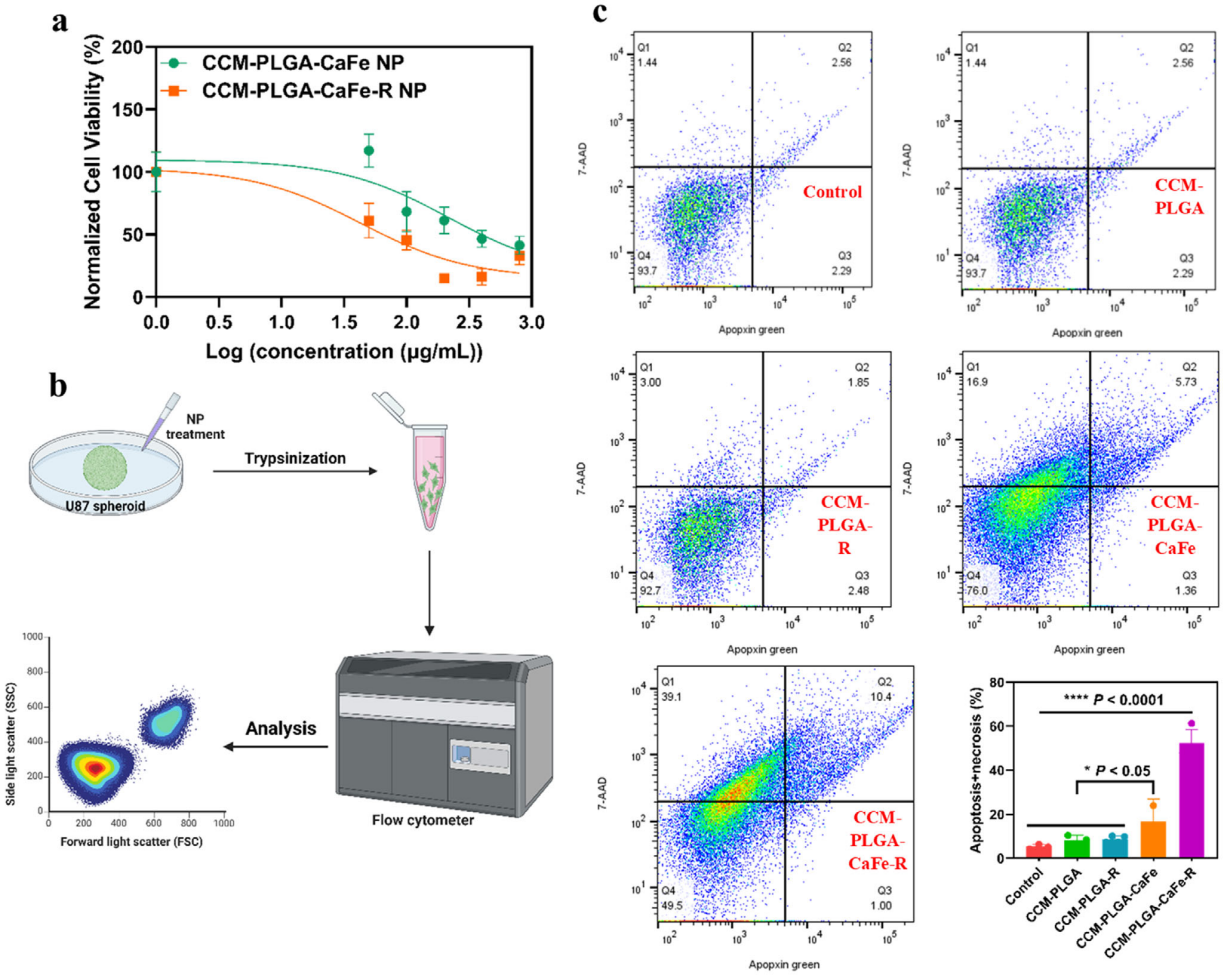
In vitro antitumor assay. a) In vitro antitumor assay of nanoparticles using GPF‐U87 spheroids b) Schematic illustration of flow cytometric analysis of apoptosis/necrosis in U87 spheroids. c) Flow cytometric analysis of U87 spheroids with different nanoparticle treatments and the corresponding apoptosis and necrosis ratios of each treatment. Statistical analysis was performed using One‐way ANOVA (*n* = 3). * *p*< 0.05, **** *p*< 0.0001.

Given the TME‐responsive nature of these nanoparticles, we further evaluated their antitumor efficacy in U87 spheroids to mimic the TME better. 3D cell spheroids closely resemble avascular tumor nodules, exhibiting similar volume growth kinetics, histomorphological features, and physicochemical gradients, such as oxygen/nutrient gradients.^[^ ^58^ ^]^ Owing to these properties, multicellular spheroids are widely employed in preclinical drug testing to better estimate in vivo antitumor efficacy. As shown in Figure S12 (Supporting Information), after 7 days, CCM‐PLGA NPs exhibited minimal impact on U87 spheroid viability up to 800 µg mL^−1^. Interestingly, CCM‐PLGA‐R NPs appeared to promote cell proliferation, likely due to the tumor growth‐promoting effects of low concentrations of NONO.^[^ ^59^ ^]^ Both CCM‐PLGA‐CaFe NPs and CCM‐PLGA‐CaFe‐R NPs displayed dose‐dependent cytotoxicity (**Figure** **8**a). Importantly, polyarginine in PLGA‐CaFe‐R NPs significantly enhanced their therapeutic efficacy, with the IC50 value shifting from 216.9 µg mL^−1^ for PLGA‐CaFe NPs to 43.38 µg mL^−1^ for PLGA‐CaFe‐R NPs. An apoptosis/necrosis assay was conducted using multiplex fluorescent staining to explore further cell death mechanisms (Figure 8b,c). Apoptotic cells were stained using Apopxin Green dye, while late apoptotic or necrotic cells were stained with 7‐AAD. Flow cytometry analysis revealed that CCM‐PLGA‐CaFe‐R NPs induced a significantly higher proportion of dead cells (sum of Q1+Q2+Q3, 52.3% ± 6.1%) than CCM‐PLGA‐CaFe NPs (16.7% ± 10.25%), CCM‐PLGA‐R NPs (8.70% ± 1.52%), CCM‐PLGA NPs (8.37% ± 2.06%), and the control (5.27% ± 1.04%). The results indicate that late apoptosis or necrosis is the predominant form of cell death induced by both PLGA‐CaFe NPs and PLGA‐CaFe‐R NPs.

## 3 Conclusion

In this study, we developed a novel, tumor microenvironment (TME)‐responsive nanoparticle platform (CCM‐PLGA‐CaFe‐R NPs) with self‐accelerating degradation for enhanced chemodynamic tumor therapy. By integrating PLGA‐encapsulated Ca‐Fe peroxide nanoparticles with polyarginine and camouflaging them with cancer cell membranes, we achieved targeted delivery, stability in physiological conditions, and specific degradation of nanoparticles within the TME. Our unique design addressed key limitations of traditional CDT, including insufficient H_2_O_2_ and slow Fenton reaction cycles. Polyarginine facilitated Fe^3+^ reduction to Fe^2+^, accelerating the Fenton reaction cycle and amplifying hydroxyl radical (•OH) generation. In vitro, studies demonstrated specific cellular uptake of CCM‐PLGA‐CaFe‐R NPs by U87 cells and deep penetration into 3D tumor spheroids. The nanoparticles also generated nitric oxide (NO) within spheroids, synergistically enhancing tumor cell apoptosis/necrosis through •OH‐mediated cell death. Further cytotoxicity assay also demonstrated the superior antitumor efficacy of CCM‐PLGA‐CaFe‐R NPs over other nanoparticles, highlighting its potential for robust and selective CDT. Overall, this TME‐responsive nanoparticle system provides a promising therapeutic strategy for tumors, leveraging the intrinsic TME features to maximize lethal ROS generation and improve tumor targetability.

## 4 Experimental Section

### Synthesis of PVP‐CaFe, PLGA‐CaFe NP, and PLGA‐CaFe‐R NP

CaFe peroxide nanoparticles were synthesized with modifications from a previous report.^[^ ^20^ ^]^ Briefly, 420 mg CaCl_2_.2H_2_O, 600 mg FeCl_2_.4H_2_O, and 15 g PVP‐10 were dissolved in 225 mL ethanol under sonication. Then 15 mL 0.8 m NH_3_.H_2_O was added under stirring, and 300 µL 30% H_2_O_2_ was slowly added. After stirring for one hour, nanoparticles were collected by centrifugation and washed several times with ethanol. The synthesized CaFe NPs were redispersed in ethanol and stored at 4 °C for further use.

PLGA was dissolved in a mixture of dichloromethane (DCM) and ethyl acetate (EA) (v/v, 3:2) at 20 mg mL^−1^. 4 mg CaFe NPs or 4 CaFe NPs + 4 mg polyarginine were dispersed in 200 µL DI water and added to 1 mL PLGA solution under vortexing. Then, the emulsion was ultrasonicated for 90 s (15 seconds on/15 seconds off, 40% amplitude). The resultant emulsion was added to 2 mL 4% PVA solution (w/v) and ultrasonicated for another 90 s. Subsequently, the emulsion was added to 8 mL 1% PVA solution (w/v) and stirred overnight. Then, PLGA‐coated nanoparticles were collected by centrifugation and washed several times with DI water. To quantify the weight content of encapsulated CaFe NPs in PLGA, thermogravimetric analysis (TGA) was performed using a Q50 Thermogravimetric Analyzer (TA Instruments).

### Cell Culture and Cell Membrane Preparation

Human glioblastoma cell line U87 (ATCC) and GFP‐expressing U87 cells (GFP‐U87, Angio‐Proteomie) were cultured in Eagle's Minimum Essential Medium (EMEM, ATCC) with 10% fetal bovine serum (FBS, Thermo Fisher Scientific) and 1% penicillin‐streptomycin (Thermo Fisher Scientific). Human brain umbilical endothelial cells (HUVECs, ScienCell) were cultured in endothelial cell medium (ECM, ScienCell) with endothelial cell growth supplement (ECGS, ScienCell) and 1% antibiotic solution (P/S, ScienCell). Cells were maintained in the incubator at 37 °C, 5% CO_2_, and 95% relative humidity until ≈90% confluence was reached. Then, cells were harvested using 0.05% EDTA‐Trypsin and collected for further assays.

U87 cells were harvested and washed with cold 1 × Dulbecco's phosphate‐buffered saline (DPBS, Thermo Fisher Scientific) three times to obtain cancer cell membranes. Afterward, cells were redispersed in the DPBS and kept on ice for 15 min. After three thaw‐freeze cycles in a ‐80 °C freezer, cell debris was centrifuged at 700× g for 10 min. The pellet was discarded, and the supernatant was centrifuged at 14000× g for 30 min. After centrifugation, the supernatant was decanted, and the pellet was redispersed in DI water. The concentration of cancer cell membranes was quantified using the Bradford assay (Bio‐Rad). The obtained cancer cell membrane was stored at −80 °C for further use.

### Synthesis of Cancer Cell Membrane‐Coated Nanoparticles

To prepare cancer cell membrane camouflaged nanoparticles, 1 mg of nanoparticles in DI water were mixed with 0.1 mg of the cancer cell membrane (CCM) in DI water and ultrasonicated for 2 min (15 s on/off, 40% amplitude). The camouflaged nanoparticles were collected by centrifugation and stored at 4 °C for further use.

### Effect of pH on Nanoparticle Degradation

Nanoparticle degradation was monitored by the oxidation of TMB and its color change. To assess the pH effects on degradation, 40 µL of nanoparticles at 1 mg/mL CaFe (prepared in 50 mM sodium acetate buffer) and 4 µL of 10 mg mL^−1^ TMB solution (dissolved in DMSO) were added to 356 µL of 50 mM sodium acetate buffer. The pH of each solution was adjusted to pH 7.4, 6.5, and 4.5 using 1 m acetic acid, followed by incubation at 37 °C for three hours. Absorbance at 652 nm was measured over time with a microplate reader (Tecan Infinite 200 Pro M Plex).

### Effect of H_2_O_2_ on Nanoparticle Degradation

40 µL of nanoparticles (1 mg mL CaFe), 4 µL of 10 mg mL^−1^ TMB, and 40 µL of 1 mm H_2_O_2_ (prepared in sodium acetate buffer) were added to 316 µL of sodium acetate buffer. The solutions were then incubated at 37 °C and the absorbance at 652 nm was monitored over time.

### H_2_O_2_‐Responsive Degradation of Nanoparticles

40 µL of nanoparticles (1 mg mL^−1^ CaFe), 4 µL of 10 mg mL^−1^ TMB or OPD solution, and 40 µL of H_2_O_2_ (0, 0.5, 1, or 5 mm) were added to 316 µL of sodium acetate buffer. The pH was adjusted to 4.5 with 1 m acetic acid, and the solutions were incubated at 37 °C. Color changes for TMB and OPD were measured at 652 nm and 417 nm, respectively. UV‐vis absorption spectra of oxidized TMB and OPD were also obtained using a microplate reader.

### Detection of •OH Production by APF and RhB

Nanoparticles (200 µg mL^−1^ CaFe) were incubated with 2.5 µm APF (prepared in DMF) and 100 µm H_2_O_2_ in sodium acetate buffer (pH 6.5) for three hours at 37 °C. Subsequently, the fluorescence spectra of oxidized APF were obtained using a microplate reader with an excitation wavelength of 455 nm and an emission wavelength range of 500–600 nm.

For the Rhodamine B (RhB) decolorization assay, nanoparticles (200 µg mL^−1^ CaFe) were incubated with five µg mL^−1^ RhB and 100 µm H_2_O_2_ at pH 4.5. After overnight incubation at 37 °C, nanoparticle solutions were centrifuged, and the UV‐vis absorption spectra of supernatants were obtained using a microplate reader.

### Evaluation of Nanoparticle Degradation by TEM and FTIR

PLGA‐CaFe‐R NPs (100 µg mL^−1^) were incubated with 100 µM H_2_O_2_ at pH 7.4 and 4.5 for one hour, and their morphology was characterized using TEM. Separately, PLGA, PLGA‐R, and CCM‐PLGA‐R NPs (100 µg mL^−1^) were incubated with 100 µm H_2_O_2_ for 3 h, and the resulting degradation products were analyzed with FTIR.

### Assessment of Fe^2+^ Availability in Nanoparticle Solutions

The presence of Fe^2+^ in solutions was evaluated using 1,10‐phenanthroline (Phen), which forms a brown‐orange complex with Fe^2+^ ions and exhibits an absorbance peak at 510 nm.

Effect of pH on Fe^2+^ availability: Nanoparticle solutions (200 µL, containing 200 µg mL^−1^ CaFe) at different pH (7.4, 6.5, and 4.5) were incubated at 37 °C for three hours. Subsequently, each nanoparticle solution was mixed with 200 µL of sodium acetate buffer and 200 µL of 1 mg mL^−1^ Phen and incubated at room temperature for 30 min. After centrifugation, the absorbance at 510 nm and UV‐vis absorption spectra were obtained using a microplate reader.

Effect of H_2_O_2_ on Fe^2+^ availability in nanoparticle solutions: Nanoparticles (200 µL, containing 200 µg mL^−1^ CaFe) at pH 6.5 or pH 4.5 were treated with different H_2_O_2_ concentrations (0, 50, 100, 500, and 1000 µM) and incubated for three h at 37 °C. Following incubation, each solution was mixed with 200 µL of sodium acetate buffer and 200 µL of 1 mg mL^−1^ Phen and incubated at room temperature for 30 min. After centrifugation, the absorbance at 510 nm and UV‐Vis absorption spectra were obtained using a microplate reader.

Conversion of Fe^3+^ to Fe^2+^ by polyarginine: Polyarginine (15 mg mL^−1^) was pre‐incubated with one mM H_2_O_2_ for two hours to generate nitric oxide (NO). Subsequently, one mM Fe^3+^ and 1 mg/mL Phen were added, and the mixture was incubated for 24 h at 37 °C. Fe^3+^ incubated with H_2_O_2_ and polyarginine incubated with H_2_O_2_ were used as control groups.

### Cellular Uptake and Homotypic Targeting

Preparation of Dual‐fluorophore‐labeled nanoparticles: to label the nanoparticle core, 10 µg Alexa Fluor 488 (AF488) in N, N‐dimethylformamide (DMF) was mixed with 0.5 mL of a 20 mg mL^−1^ PLGA solution, before the synthesis of PLGA‐CaFe‐R NPs. The resulting PLGA‐CaFe‐R‐AF488 NPs were collected by centrifugation, washed with DI water, and stored at 4 °C.

To prepare CCM‐coated, DiD‐labeled nanoparticles, 1 mg of DiD‐labeled NPs was mixed with 100 µg of CCM, followed by centrifugation and washing with DI water. Then, 2 µg of DiD dye was added to the CCM‐PLGA‐CaFe‐R nanoparticles and sonicated for 30 min. After washing with DPBS, DiD‐CCM‐PLGA‐CaFe‐R‐AF488 nanoparticles were collected by centrifugation.

Preparation of DiD‐labeled nanoparticles: 20 µg DiD solution was added to 1 mL of 20 mg mL^−1^ PLGA solution (2 µg DiD per 1 mg PLGA) and mixed well before the synthesis of PLGA‐CaFe NPs and PLGA‐CaFe‐R NPs. The resulting PLGA‐CaFe‐DiD and PLGA‐CaFe‐R‐DiD NPs were collected by centrifugation, washed with DI water, and stored at 4 °C. To prepare CCM‐coated DiD‐labeled NPs, 1 mg of DiD‐labeled NPs was mixed with 100 µg of CCM, followed by centrifugation and washing with DI water.

CCM integrity assay: U87 cells were seeded in a 96‐well plate (10000 cells per well) and incubated overnight. Subsequently, 100 µg mL^−1^ dual‐fluorophore‐label nanoparticles were added to each well and incubated for 2 h. After incubation, cells were washed with DPBS three times, fixed with 4% paraformaldehyde, and stained with 10 µg mL^−1^ DAPI for nuclear visualization. Fluorescent images were acquired using a Lionheart automated microscope.

Homotypic targeting assay: U87 cells and HUVECs (5 × 10^5^ each) were seeded in 35 mm Petri dishes and incubated overnight for attachment. To assess CCM's effect on tumor targetability, U87 cells were treated with 10 µg/mL DiD‐labeled NPs or CCM‐DiD‐labeled NPs. For tumor selectivity, 10 µg mL^−1^ CCM‐DiD‐labeled NPs were added to U87 cells and HUVECs. After 4 h of incubation, cells were washed with DPBS, detached with EDTA‐Trypsin solution, collected via centrifugation, and analyzed using an Amnis ImageStream flow cytometer.

Subcellular distribution: U87 cells (10000 per well) were seeded in a 96‐well glass‐bottom plate (Cellvis). After 24 h, cells were treated with 10 µg mL^−1^ CCM‐PLGA‐CaFe‐R‐DiD NPs for 0.5, 2, or 4 h. Subsequently, cells were stained with DAPI (10 µg mL^−1^) and LysoBrite Green (5 µg mL^−1^) for 30 min at 37 °C. After the incubation, cells were washed three times with DPBS and imaged using a fluorescent microscope (Biotek Lionheart LX Automated Microscope). Colocalization of lysosomes and DiD dye was analyzed with the JACoP plugin in Image J software (NIH).

### Tumor Spheroid Penetration

U87 cells were seeded in a 96‐well ultra‐low attachment plate (S‐Bio) at 10 000 cells per well and incubated for 3 days to form spheroids (>400 µm diameter). CCM‐DiD‐labeled nanoparticles (10 µg mL^−1^) were added and incubated overnight. Spheroids were washed thrice with DPBS, fixed with 4% paraformaldehyde, and stained with DAPI at room temperature for 1 h. Nanoparticle penetration was analyzed using laser‐scanning confocal microscopy (LSCM, ImageXpress, Molecular Devices), and the penetration depth was quantified with Image J (NIH).

### Hypoxia and NO Staining in Tumor Spheroid

Cancer spheroids were fabricated and incubated with nanoparticles (100 µg mL^−1^ CaFe) overnight. After washing with DPBS, hypoxia and NO were stained with 20 µm ImageIT Green (Fisher Scientific) and DAR‐1 (Sigma Aldrich). LSCM obtained the fluorescent images, and fluorescent intensity was analyzed using ImageJ software (NIH).

### Intracellular Detection of Total ROS and Hydroxyl Radicals

U87 spheroids were prepared using the above method. Nanoparticles (100 µg mL^−1^ of CaFe) were incubated with spheroids for 24 h. Subsequently, spheroids were washed with DPBS three times and stained with 20 µm DCHF‐DA probe and 10 µg mL^−1^ DAPI for 30 min at room temperature. Similarly, •OH was stained by 20 µm APF at room temperature for 30 min. The fluorescent images of spheroids were obtained by LSCM and quantified by ImageJ software.

### In Vitro Anti‐tumor Efficacy

For short‐term cytotoxicity assay, 10 000 GFP‐U87 cells were seeded into a 96‐well plate with black walls and clear bottom (Corning, 3610) and incubated in a 37 °C incubator for 24 h. Then, the cell culture medium was replaced with nanoparticle solutions. After another 24 h of incubation, cells were washed with DPBS and imaged by LSCM. ImageJ analyzed the green fluorescence of cells.

For the in vitro antitumor assay,10 000 GFP‐U87 cells were seeded into an ultra‐low attachment plate and incubated for 3 days to form large‐diameter spheroids. Nanoparticles were added to each well, and the treatment medium was changed every two days. After 7 days, spheroids were imaged by LSCM, the green fluorescence was quantified by ImageJ and cell viability was normalized by the green fluorescence of the control group.

### In Vitro Apoptosis/Necrosis Assay

Large U87 spheroids were formed using the above method and treated with nanoparticles (200 µg mL^−1^ CaFe) for 48 h. After washing with DPBS, spheroids were dissociated with a trypsin‐EDTA solution at 37 °C for 10 min. After washing, cells were stained with an apoptotic and necrotic detection kit for 30 min at room temperature and immediately analyzed by a flow cytometer (Luminex ImageStream). The apoptosis and necrosis ratios of treated cells were analyzed and quantified by FlowJo 10.8 software.

### Statistical Analysis

All experiments were performed in at least triplets. All data were presented in Mean ± Standard Deviation, and statistical comparisons were performed using one‐sided Student's *t*‐test or one‐way ANOVA with an alpha value of 0.05. *p* value <0.05 was considered statistically significant, with specific significance levels denoted as follows: *p < 0.05, **p < 0.01, ***p < 0.001, and ****p < 0.0001. All data were analyzed using GraphPad Prism 9.

## Conflict of Interest

The authors declare no conflict of interest.

## Supporting information



Supporting Information

## Data Availability

The data that support the findings of this study are available from the corresponding author upon reasonable request.

## References

[1] R. A. Cairns , I. S. Harris , T. W. Mak , Nat. Rev. Cancer 2011, 11, 85. 21258394 10.1038/nrc2981

[2] J. D. Hayes , A. T. Dinkova‐Kostova , K. D. Tew , Cancer Cell 2020, 38, 167. 32649885 10.1016/j.ccell.2020.06.001 PMC7439808

[3] P. T. Schumacker , Cancer Cell 2006, 10, 175. 16959608 10.1016/j.ccr.2006.08.015

[4] A. Glasauer , N. S. Chandel , Biochem. Pharmacol. 2014, 92, 90. 25078786 10.1016/j.bcp.2014.07.017

[5] W. Du , T. Liu , F. Xue , X. Cai , Q. Chen , Y. Zheng , H. Chen , ACS Appl. Mater. Interfaces 2020, 12, 19285. 32249558 10.1021/acsami.0c02465

[6] M. Huo , L. Wang , Y. Chen , J. Shi , Nat. Commun. 2017, 8, 357. 28842577 10.1038/s41467-017-00424-8 PMC5572465

[7] W. Sun , C. Zhu , J. Song , S. C. Ji , B. P. Jiang , H. Liang , X. C. Shen , Adv. Healthcare Mater. 2023, 12, 2300385. 10.1002/adhm.202300385 37040018

[8] S. Xiao , Y. Lu , M. Feng , M. Dong , Z. Cao , X. Zhang , Y. Chen , J. Liu , Chem. Eng. J. 2020, 396, 125294.

[9] X. Meng , D. Li , L. Chen , H. He , Q. Wang , C. Hong , J. He , X. Gao , Y. Yang , B. Jiang , ACS Nano 2021, 15, 5735. 33705663 10.1021/acsnano.1c01248

[10] Y. Liu , W. Zhen , Y. Wang , J. Liu , L. Jin , T. Zhang , S. Zhang , Y. Zhao , S. Song , C. Li , Angew. Chem. 2019, 131, 2429. 10.1002/anie.201813702 30600877

[11] Z. Yu , Y. Hu , Y. Sun , T. Sun , Chem.‐Eur. J. 2021, 27, 13953. 34196066 10.1002/chem.202101514

[12] Q. Chen , D. Yang , L. Yu , X. Jing , Y. Chen , Mater. Horiz. 2020, 7, 317.

[13] Y. Zhou , S. Fan , L. Feng , X. Huang , X. Chen , Adv. Mater. 2021, 33, 2104223. 10.1002/adma.202104223 34580933

[14] C. Korupalli , C.‐C. Kuo , G. Getachew , W. B. Dirersa , A. Wibrianto , A. S. Rasal , J.‐Y. Chang , J. Colloid Interface Sci. 2023, 643, 373. 37080044 10.1016/j.jcis.2023.04.049

[15] W. B. Dirersa , G. Getachew , A. Wibrianto , A. S. Rasal , V. S. Gurav , M. Z. Fahmi , J.‐Y. Chang , J. Colloid Interface Sci. 2023, 647, 528. 37230831 10.1016/j.jcis.2023.05.099

[16] D. Jana , B. He , Y. Chen , J. Liu , Y. Zhao , Adv. Mater. 2024, 36, 2206401. 10.1002/adma.202206401 36210733

[17] G. G. Demissie , Y.‐C. Chen , S.‐Y. Ciou , S.‐H. Hsu , C.‐Y. Wang , C.‐C. Huang , H.‐T. Chang , Y.‐C. Lee , J.‐Y. Chang , J. Colloid Interface Sci. 2025, 685, 396. 39855086 10.1016/j.jcis.2025.01.149

[18] G. Getachew , C. Korupalli , A. S. Rasal , J.‐Y. Chang , Composites, Part B 2021, 226, 109364.

[19] L.‐S. Lin , T. Huang , J. Song , X.‐Y. Ou , Z. Wang , H. Deng , R. Tian , Y. Liu , J.‐F. Wang , Y. Liu , J. Am. Chem. Soc. 2019, 141, 9937. 31199131 10.1021/jacs.9b03457

[20] S. Shen , M. Mamat , S. Zhang , J. Cao , Z. D. Hood , L. Figueroa‐Cosme , Y. Xia , Small 2019, 15, 1902118. 10.1002/smll.201902118 31328882

[21] S. Dong , Y. Dong , B. Liu , J. Liu , S. Liu , Z. Zhao , W. Li , B. Tian , R. Zhao , F. He , Adv. Mater. 2022, 34, 2107054. 10.1002/adma.202107054 34865269

[22] S. Koo , O. K. Park , J. Kim , S. I. Han , T. Y. Yoo , N. Lee , Y. G. Kim , H. Kim , C. Lim , J.‐S. Bae , ACS Nano 2022, 16, 2535. 35080370 10.1021/acsnano.1c09171

[23] B. Liu , Y. Bian , S. Liang , M. Yuan , S. Dong , F. He , S. Gai , P. Yang , Z. Cheng , J. Lin , ACS Nano 2021, 16, 617. 34957819 10.1021/acsnano.1c07893

[24] W. B. Dirersa , T.‐C. Kan , J. Chang , G. Getachew , S. Ochirbat , S. Kizhepat , A. Wibrianto , A. Rasal , H.‐A. Chen , A. V. Ghule , ACS Appl. Mater. Interfaces 2024, 16, 24172. 38688027 10.1021/acsami.3c18888 PMC11103653

[25] L. Zhang , S.‐S. Wan , C.‐X. Li , L. Xu , H. Cheng , X.‐Z. Zhang , Nano Lett. 2018, 18, 7609. 30383966 10.1021/acs.nanolett.8b03178

[26] Y. Guo , H. R. Jia , X. Zhang , X. Zhang , Q. Sun , S. Z. Wang , J. Zhao , F. G. Wu , Small 2020, 16, 2000897. 10.1002/smll.202000897 32537936

[27] F. Chen , B. Yang , L. Xu , J. Yang , J. Li , ChemMedChem 2021, 16, 2278. 33792182 10.1002/cmdc.202100108

[28] İ. Gülçin , Z. Huyut , M. Elmastaş , H. Y. Aboul‐Enien , Arabian J. Chem. 2010, 3, 43.

[29] G. K. Lopes , H. M. Schulman , M. Hermes‐Lima , Bioch. Biophys. Acta 1999, 1472, 142. 10.1016/s0304-4165(99)00117-8 10572935

[30] Y. C. Li , L. G. Bachas , D. Bhattacharyya , Environ. Eng. Sci. 2005, 22, 756.

[31] J. Tejero , S. Basu , C. Helms , N. Hogg , S. B. King , D. B. Kim‐Shapiro , M. T. Gladwin , J. Biol. Chem. 2012, 287, 18262. 22493289 10.1074/jbc.M111.298927 PMC3365727

[32] R. Farias‐Eisner , G. Chaudhuri , E. Aeberhard , J. M. Fukuto , J. Biol. Chem. 1996, 271, 6144. 8626402 10.1074/jbc.271.11.6144

[33] A. N. Woodmansee , J. A. Imlay , Mol. Microbiol. 2003, 49, 11. 12823807 10.1046/j.1365-2958.2003.03530.x

[34] P. Agashe , A. Kuzminov , J. Biol. Chem. 2022, 298. 10.1016/j.jbc.2022.101825 PMC9018393 35288189

[35] P. Agashe , A. Kuzminov , Genetics 2021, 218, 101825. 10.1093/genetics/iyab057 PMC8225348 34027548

[36] A. Wijaya , Y. Wang , D. Tang , Y. Zhong , B. Liu , M. Yan , Q. Jiu , W. Wu , G. Wang , J. Mater. Chem. B 2022, 10, 607. 34994373 10.1039/d1tb01455b

[37] L. Liu , X. Bai , M.‐V. Martikainen , A. Kårlund , M. Roponen , W. Xu , G. Hu , E. Tasciotti , V.‐P. Lehto , Nat. Commun. 2021, 12, 5726. 34593813 10.1038/s41467-021-26052-x PMC8484581

[38] W. Yan , X. Zhou , S. Wang , J. Qiu , J. Neuromorphic Intell. 2025, 2, 1.

[39] L. Liu , W. Yu , J. Seitsonen , W. Xu , V. P. Lehto , Chem.‐Eur. J. 2022, 28, 202200947. 10.1002/chem.202200947 PMC10091812 36116117

[40] S. Chaki , T. J. Malek , M. Chaudhary , J. Tailor , M. Deshpande , Adv. Nat. Sci. Nanosci. Nanotechnol. 2015, 6, 035009.

[41] T. Dedecan , N. Baylan , İ. İnci , Chem. Phys. Lett. 2022, 797, 139581.

[42] R. Qin , L. Hao , J. Li , J. Inorg. Organomet. Polym. Mater. 2020, 30, 4452. 10.1007/s10904-020-01555-0 PMC7189358 32351348

[43] J.‐C. Wang , J. Ren , H.‐C. Yao , L. Zhang , J.‐S. Wang , S.‐Q. Zang , L.‐F. Han , Z.‐J. Li , J. Hazard. Mater. 2016, 311, 11. 26954471 10.1016/j.jhazmat.2016.02.055

[44] Z. Wang , Y. Zhang , Z. Tan , Q. Li , Chem. Eng. J. 2018, 350, 767.

[45] H. Zhou , X. Li , D. Niu , Y. Li , X. Liu , C. Li , W. Si , J. Cao , Y. Song , G. Wen , Adv. Healthcare Mater. 2021, 10, 2002126. 10.1002/adhm.202002126 33644985

[46] R. Al‐Gaashani , S. Radiman , N. Tabet , A. Daud , J. Alloys Compd. 2013, 550, 395.

[47] S. Gao , Y. Jin , K. Ge , Z. Li , H. Liu , X. Dai , Y. Zhang , S. Chen , X. Liang , J. Zhang , Adv. Sci. 2019, 6, 1902137. 10.1002/advs.201902137 PMC6918120 31871871

[48] W. Yan , J. Qiu , J. Neuromorphic Intell. 2024, 1, 9.

[49] F. Gong , N. Yang , X. Wang , Q. Zhao , Q. Chen , Z. Liu , L. Cheng , Nano Today 2020, 32, 100851.

[50] S. C. J. Loo , C. P. Ooi , Y. C. F. Boey , Polym. Degrad. Stab. 2004, 83, 259.

[51] A. Shibata , S. Yada , M. Terakawa , Sci. Rep. 2016, 6, 27884. 27301578 10.1038/srep27884 PMC4908658

[52] Z. Zhang , X. Wang , R. Zhu , Y. Wang , B. Li , Y. Ma , Y. Yin , Polym. Sci. Ser. B 2016, 58, 720.

[53] R. H. Fang , C.‐M. J. Hu , B. T. Luk , W. Gao , J. A. Copp , Y. Tai , D. E. O'Connor , L. Zhang , Nano Lett. 2014, 14, 2181. 24673373 10.1021/nl500618u PMC3985711

[54] C.‐M. J. Hu , L. Zhang , S. Aryal , C. Cheung , R. H. Fang , L. Zhang , Proc. Natl. Acad. Sci. USA 2011, 108, 10980. 21690347 10.1073/pnas.1106634108 PMC3131364

[55] R. Z. Lin , H. Y. Chang , Biotechnol. J. 2008, 3, 1172.18566957

[56] G. Lazzari , P. Couvreur , S. Mura , Polym. Chem. 2017, 8, 4947.

[57] A. I. Minchinton , I. F. Tannock , Nat. Rev. Cancer 2006, 6, 583. 16862189 10.1038/nrc1893

[58] J. Friedrich , C. Seidel , R. Ebner , L. A. Kunz‐Schughart , Nat. Protoc. 2009, 4, 309. 19214182 10.1038/nprot.2008.226

[59] W. Xu , L. Z. Liu , M. Loizidou , M. Ahmed , I. G. Charles , Cell Res. 2002, 12, 311. 12528889 10.1038/sj.cr.7290133

