# Supplementary material for: Tumor Microenvironment‐responsive Nanocatalyst for Targeted Chemodynamic Cancer Therapy
Source: Adv Healthc Mater. 2025 Jun 17;14(22):2501746. doi: 10.1002/adhm.202501746 (PMC12391643; doi:10.1002/adhm.202501746)
Supplement: Supplementary file 1 — Supporting Information [file ADHM-14-0-s001.docx]

**Supporting Information**

**Materials**

Poly(D,L-lactide-*co*-glycolide) (PLGA, Resomer RG 653H, acid terminated, Mw 24,000-38,000), calcium chloride dihydrate (CaCl_2_.2H_2_O), iron (II) chloride tetrahydrate (FeCl_2_.4H_2_O), polyvinylpyrrolidone (PVP, average Mw 10,000), poly(vinyl alcohol) (PVA, 87%-90% hydrolyzed, average Mw 30,000-70,000), 30% H_2_O_2_, 28%-30% ammonia solution, sodium acetate, 3,3’5,5’-tetramethylbenzidine (TMB), o-phenylenediamine (OPD), 1,10-phenanthroline chloride, Rhodamine B, potassium permanganate, 2’,7’-dichlorofluorescein diacetate (DCFH-DA), and DAR-1 were purchased from Sigma Aldrich. Poly (L-arginine hydrochloride) with molecular weight of 5,800 Da was ordered from Alamanda Polymers. 2-[6-(4-aminophenoxy)-3-oxo-3H-xanthen-9-yl]-benzoic acid (APF) was obtained from Cayman Chemical. Image-iT Green Hypoxia Reagent, 4’6-diamidino-2-phenylindole (DAPI) and 4% paraformaldehyde solution were ordered from Thermo Fisher Scientfic. 1,1′-dioctadecyl-3,3,3′,3′- tetramethylindodicarbocyanine, 4-chlorobenzene sulfonate salt (DiD), LysoBrite Green, and Cell Meter Apoptotic and Necrotic Multiplexing Detection Kit I were obtained from AAT Bioquest.

**Instrumentation and Characterization**

The hydrodynamic size of nanoparticles was measured by ZetaSizer (Malvern Panalytical) in DPBS. For transmission electron microscopy (TEM) characterization, all images were taken by FEI Morgagni 260. Nanoparticles were dispersed in DI water, and 5 µL solution was drop-casted on the TEM grid. For negative staining, nanoparticles were drop-casted on a TEM grid, and after 10 min, the remaining solution was removed by filter paper. Subsequently, 5 µL with 1% phosphotungstic acid with neutral pH was drop-casted on the grid. After another 10 min, the remaining solution was removed by filter paper and the grids were left to dry at room temperature overnight. For UV-Vis spectra and absorbance measurement, a microplate reader (Tecan Infinite 200 Pro M Plex) was used. Fourier Transform Infrared Spectroscopy (FTIR) analysis was performed by Bruker Alpha-Platinum. The X-ray photoelectron spectroscopy (XPS) analysis was conducted by the Omicron ESCA and the X-ray diffraction (XRD) analysis was performed using Bruker D8 X-ray.


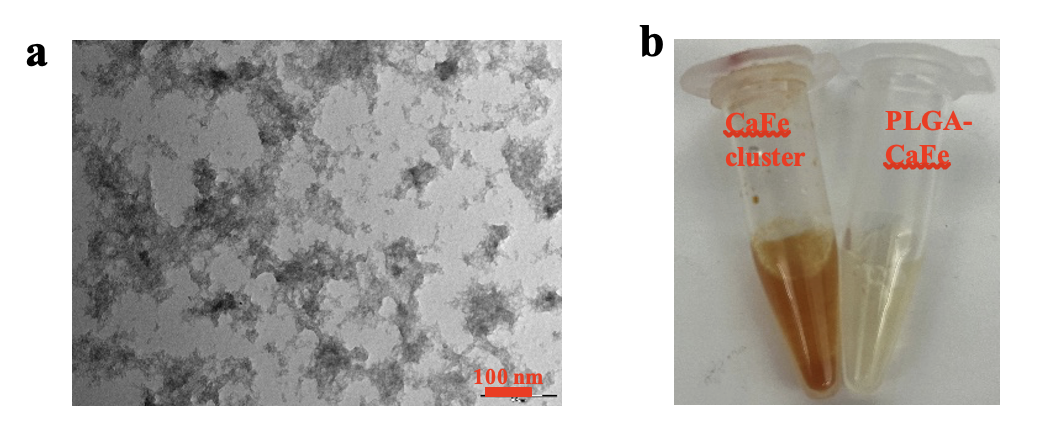


Figure S1. Characterizations of synthesized nanoparticles: a) TEM images of CaFe clusters and b) digital photographs of CaFe clusters and PLGA-CaFe in DI water after 7 days.


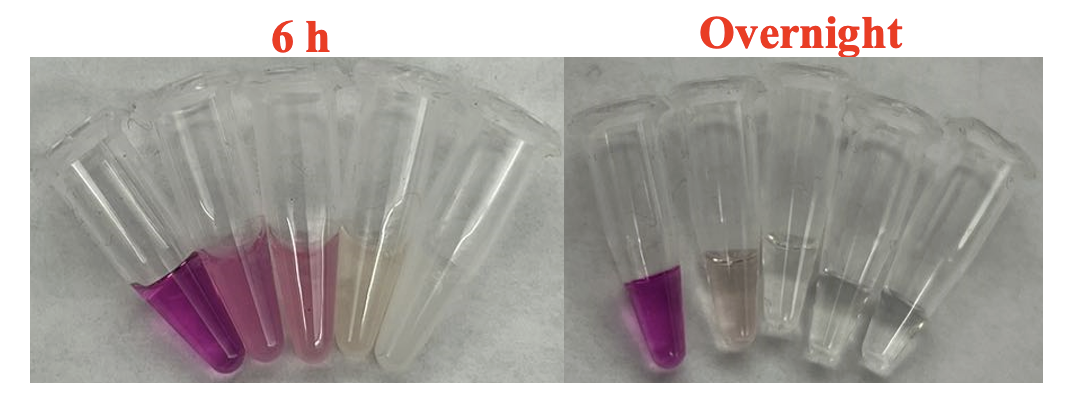


Figure S2. The colorimetric detection of peroxo groups in CaFe clusters using a KMnO4 solution. After 6 hours and overnight incubation with 0.2 mM KMnO4 under acidic conditions, various concentrations of CaFe clusters were tested (from left to right: 0 µg/mL, 100 µg/mL, 200 µg/mL, 400 µg/mL, 1000 µg/mL).


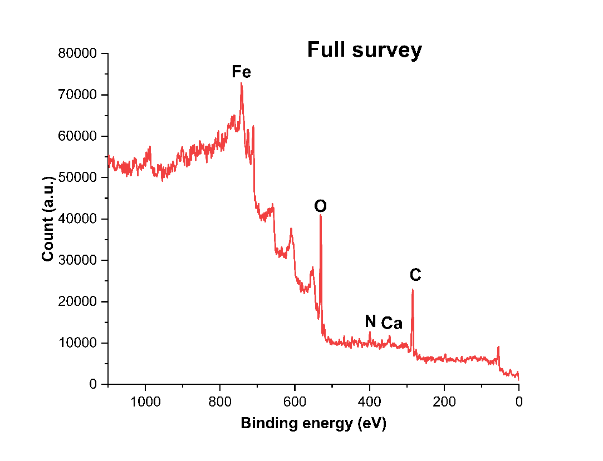


**Figure S3.** The full XPS survey scan of CaFe clusters.





**Figure S4.** TGA analysis of various nanoparticles. Temperature range:0-800 ℃; ramp rate: 10 ℃/min.


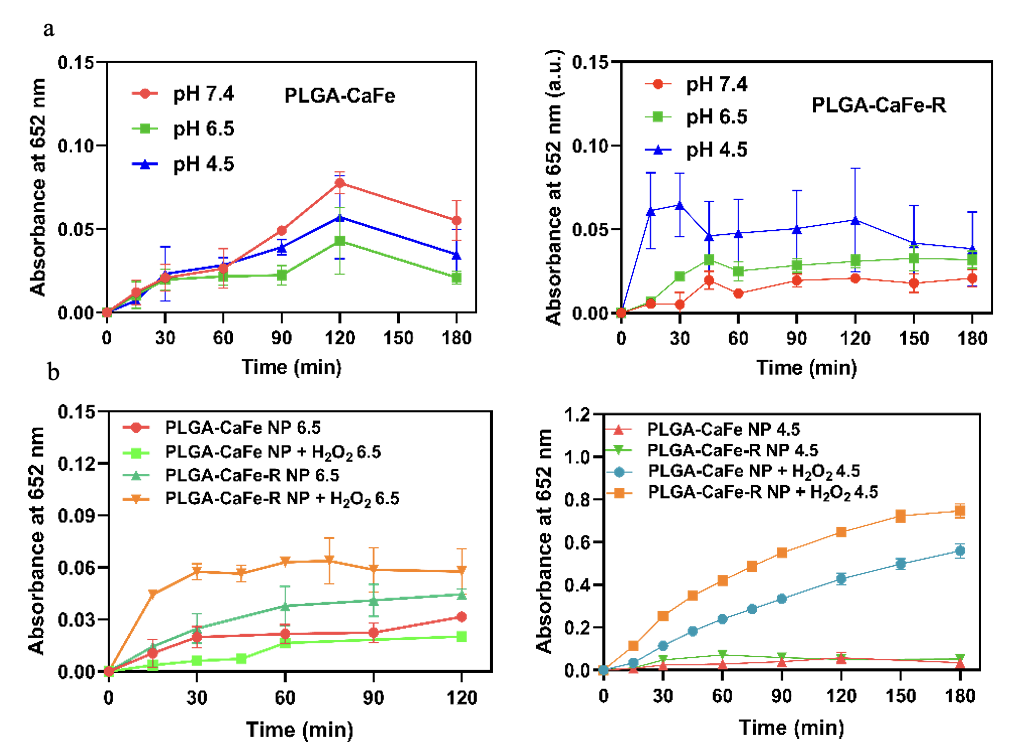


**Figure S5.** Effects of pH (a) and H_2_O_2_ (b) on the degradation-catalyzed oxidation of TMB by PLGA-CaFe NPs and PLGA-CaFe-R NPs.


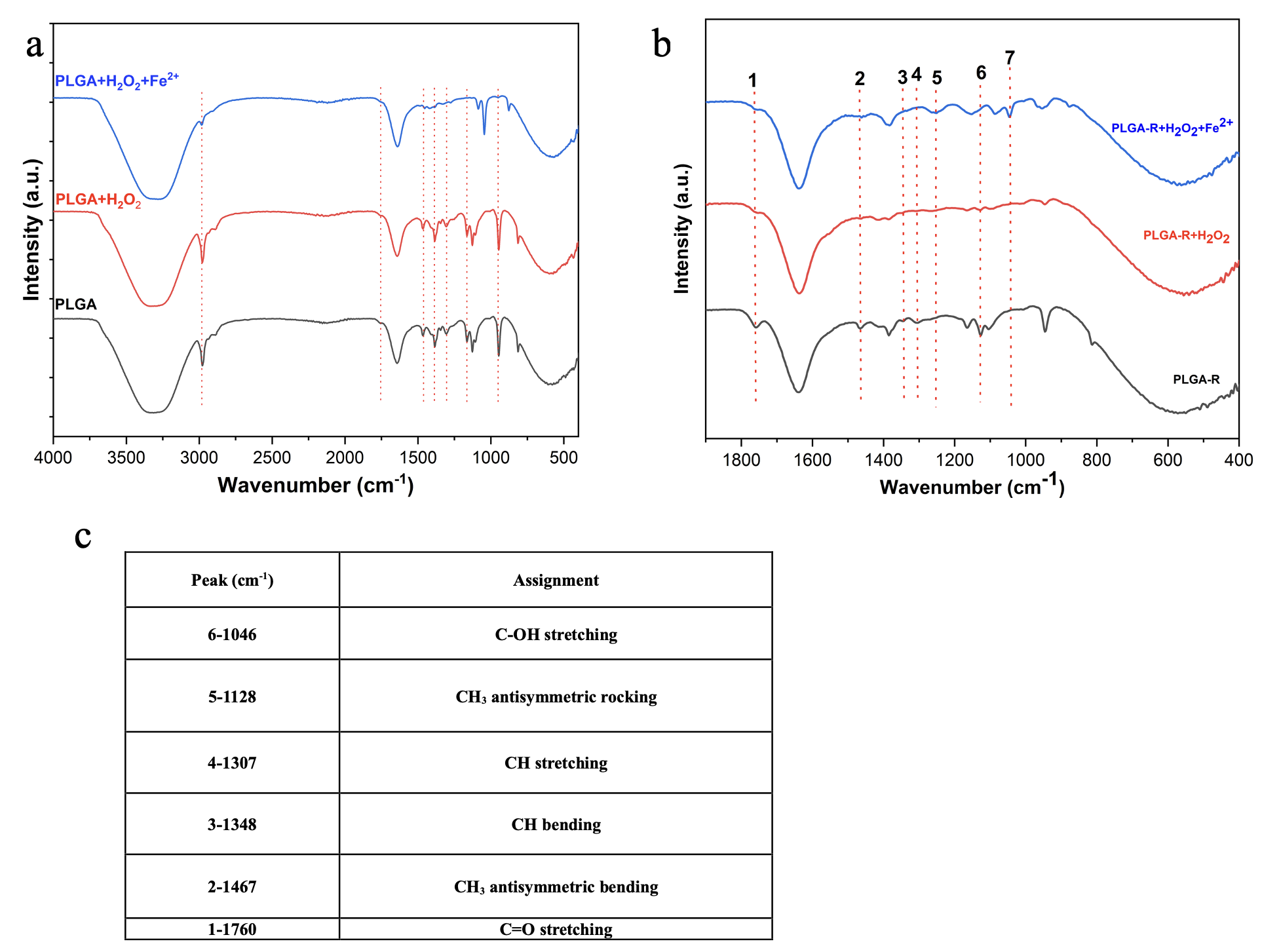


**Figure S6.** FTIR analysis of PLGA (a) and PLGA-R NPs (b) with the presence of 100 µM H_2_O_2_ and disappearance/appearance of characteristic peaks (c).


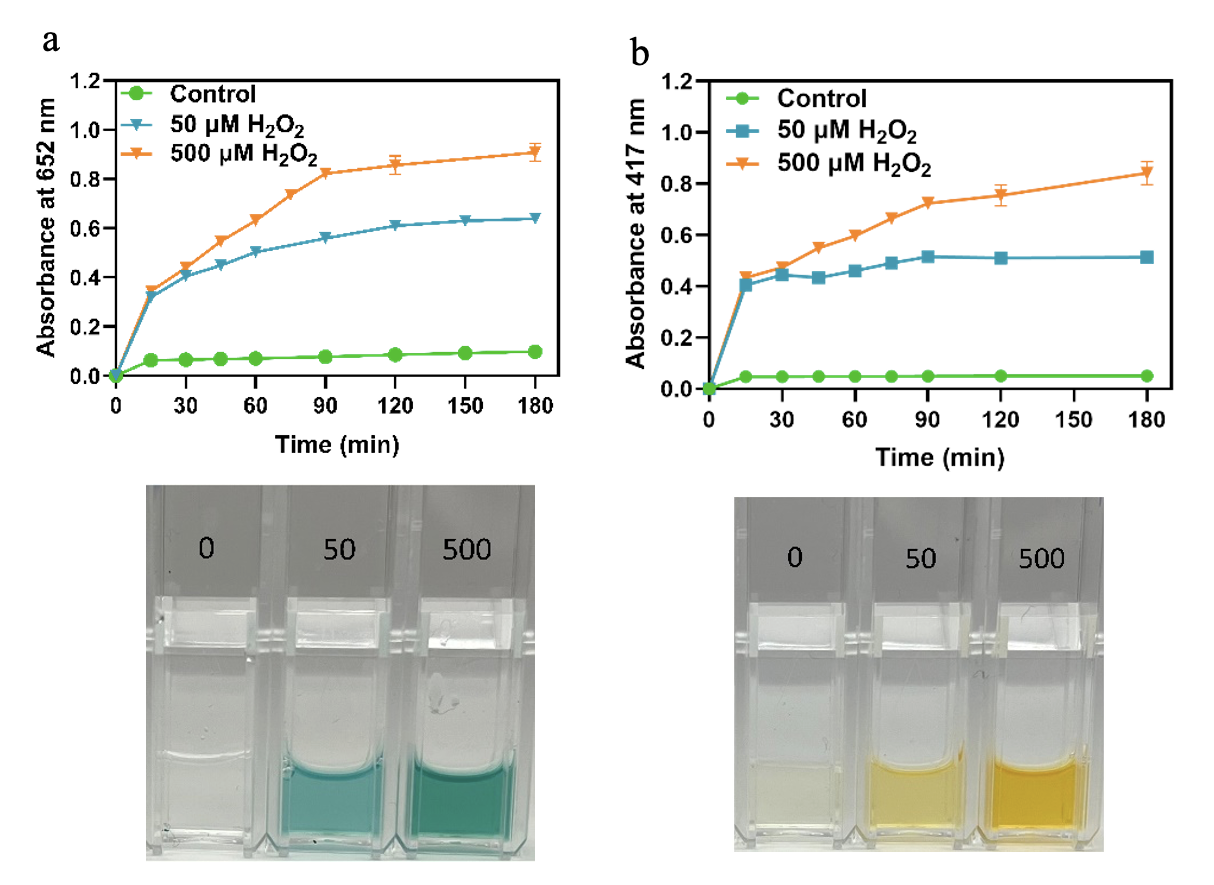


**Figure S7.** Evaluation of H_2_O_2_ responsiveness of PLGA-CaFe-R NPs using TMB (a) and OPD (b) at pH 4.5. Below are the photographs of the oxTMB and oxOPD.


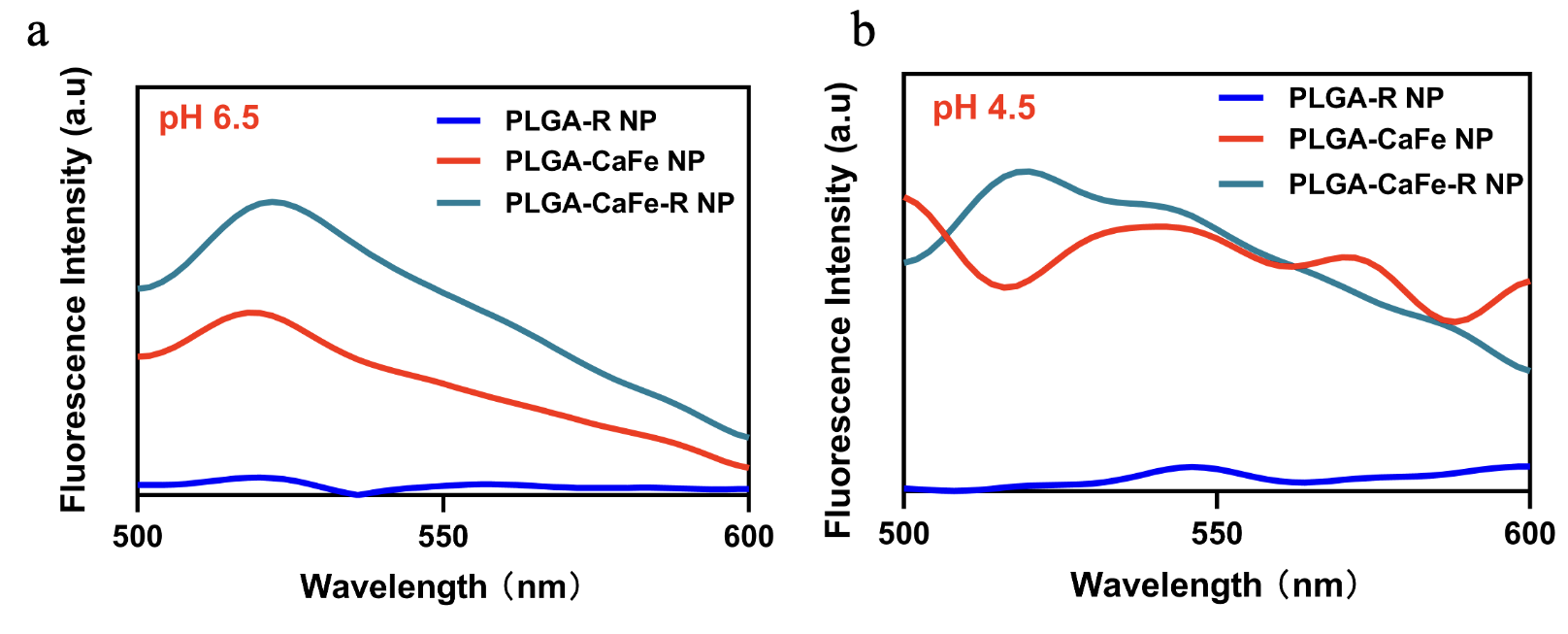


**Figure S8.** Assessment of **•**OH production of nanoparticles using APF at pH 6.5 (a) and pH 4.5 (b).


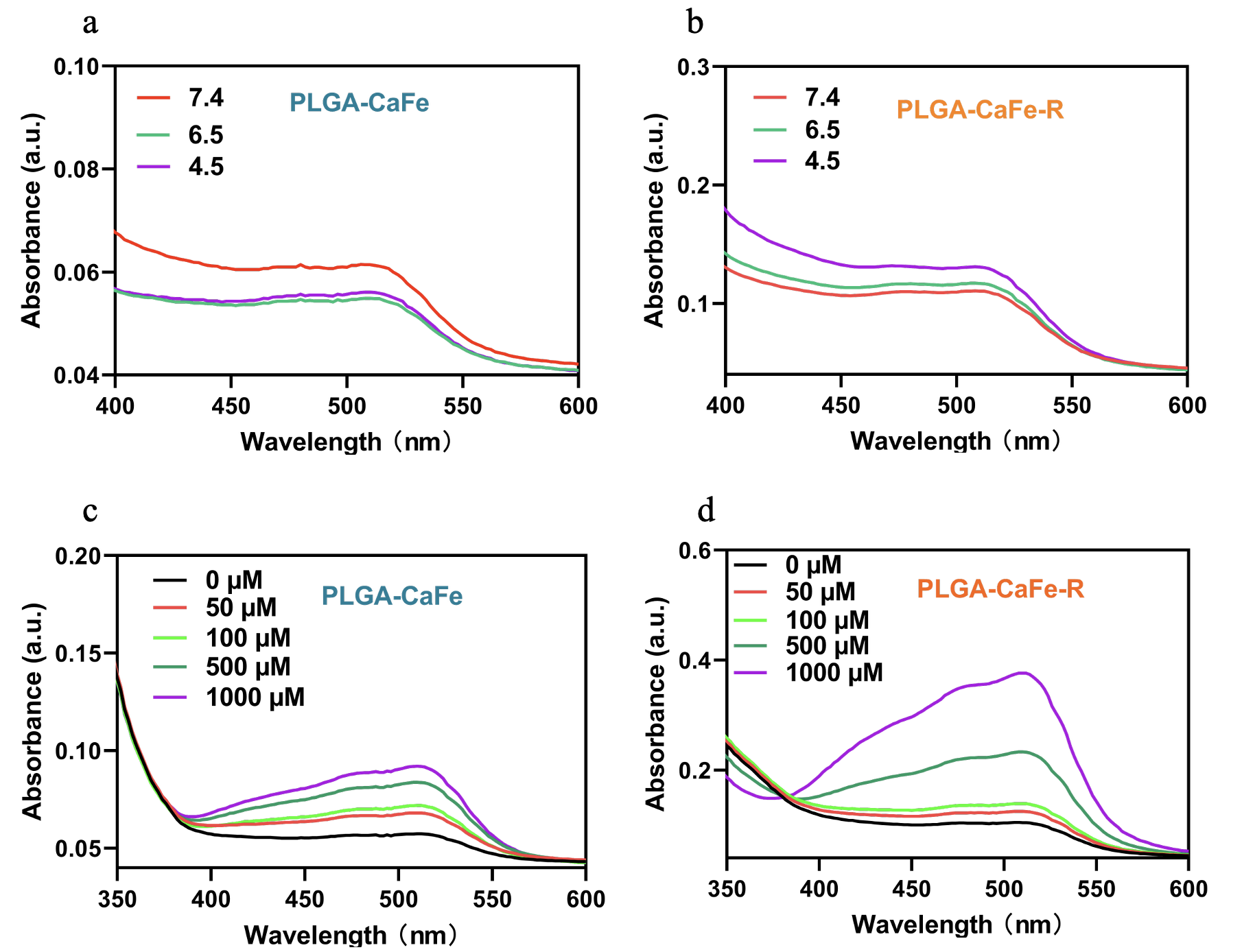


**Figure S9.** Effects of pH (a and b) and H_2_O_2_ concentrations (c and d) on Fe^2+^ availability in nanoparticles using colorimetric assay.


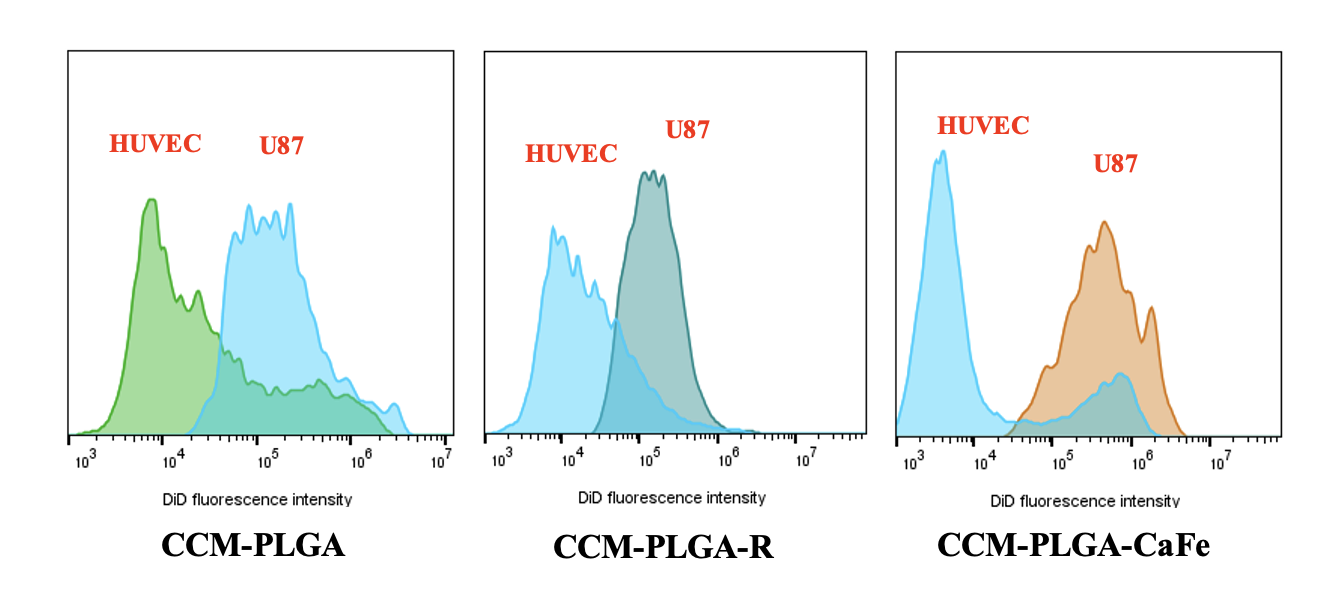


**Figure S10.** Flow cytometric analysis of the cellular uptake of DiD-labelled, CCM-PLGA, CCM-PLGA-R, and CCM-PLGA-CaFe in U87 cell and HUVECs.


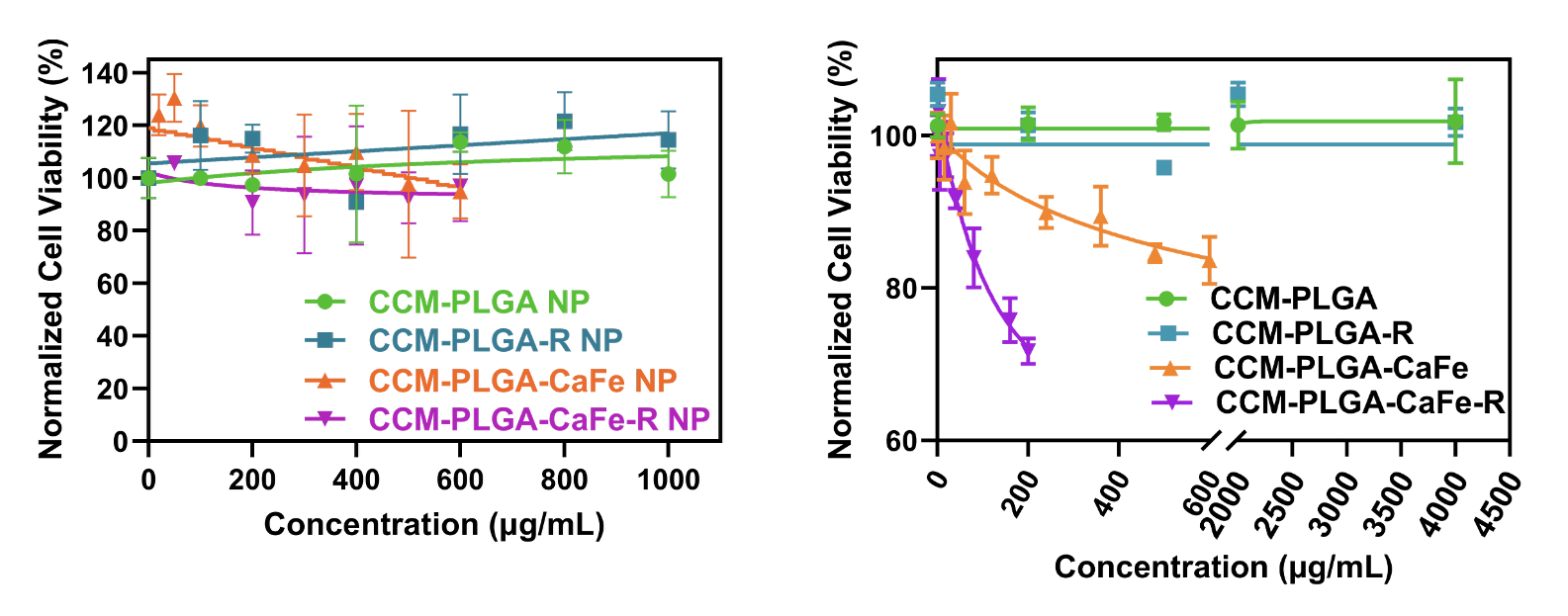


**Figure S11.** In vitro cytotoxicity of nanoparticles in RFP-HUVECs (left) and GFP-U87 cells (right). Cells were incubated with nanoparticles for 24 h and cell viability was normalized by RPF and GFP fluorescence intensity, respectively.


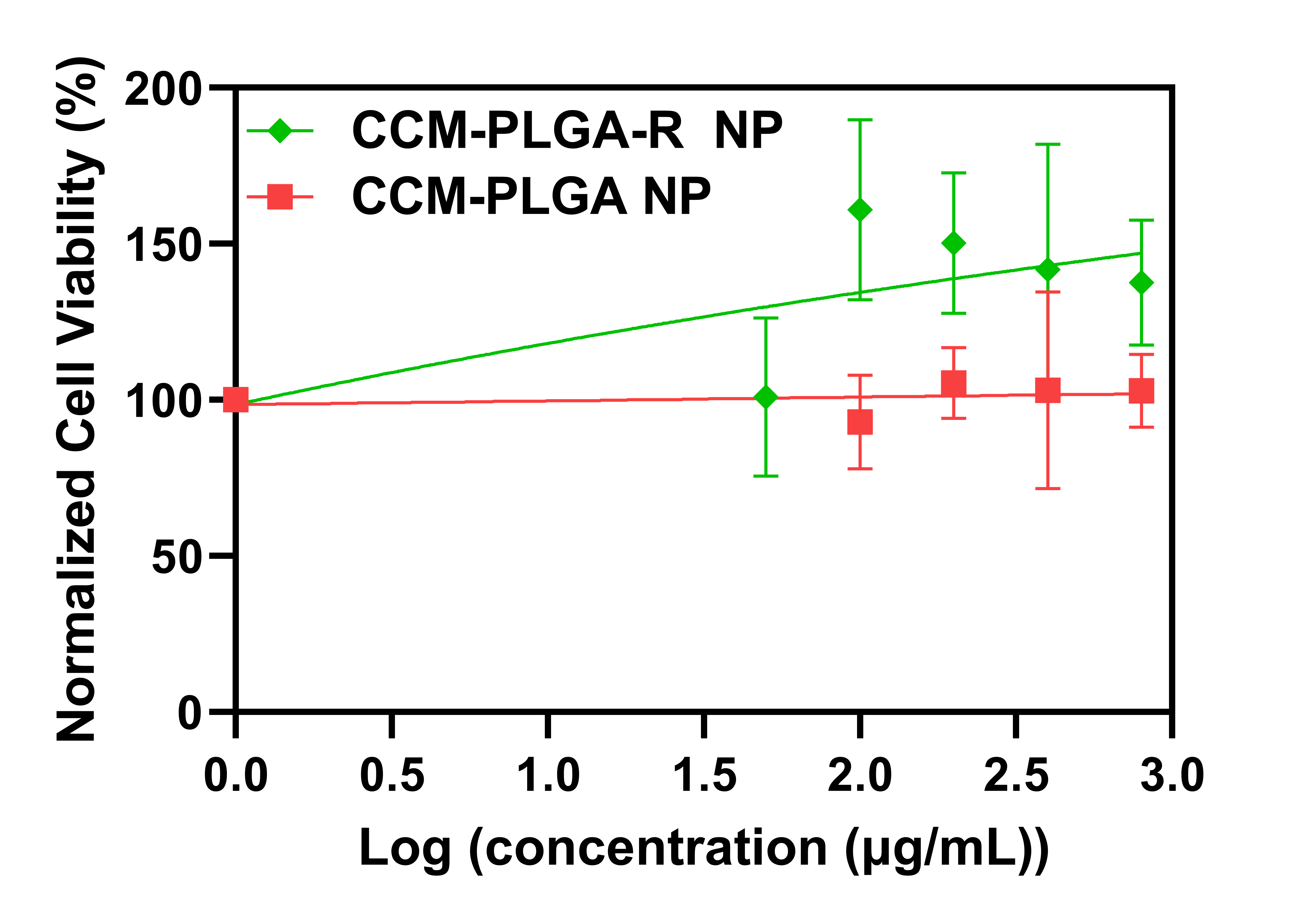


**Figure S12.** In vitro antitumor assay of CCM-PLGA and CCM-PLGA-R NPs in U87 spheroids. Spheroids were incubated with nanoparticles for 7 days and cell viability was normalized by GFP fluorescence intensity.
